# Reconstruction of the personal information from human genome reads in gut metagenome sequencing data

**DOI:** 10.1038/s41564-023-01381-3

**Published:** 2023-05-15

**Authors:** Yoshihiko Tomofuji, Kyuto Sonehara, Toshihiro Kishikawa, Yuichi Maeda, Kotaro Ogawa, Shuhei Kawabata, Takuro Nii, Tatsusada Okuno, Eri Oguro-Igashira, Makoto Kinoshita, Masatoshi Takagaki, Kenichi Yamamoto, Takashi Kurakawa, Mayu Yagita-Sakamaki, Akiko Hosokawa, Daisuke Motooka, Yuki Matsumoto, Hidetoshi Matsuoka, Maiko Yoshimura, Shiro Ohshima, Shota Nakamura, Hidenori Inohara, Haruhiko Kishima, Hideki Mochizuki, Kiyoshi Takeda, Atsushi Kumanogoh, Yukinori Okada

**Affiliations:** 1 Department of Statistical Genetics, Graduate School of Medicine, Osaka University, Suita, Japan; 2 Integrated Frontier Research for Medical Science Division, Institute for Open and Transdisciplinary Research Initiatives, Osaka University, Suita, Japan; 3 Laboratory for Systems Genetics, RIKEN Center for Integrative Medical Sciences, Yokohama, Japan; 4 Department of Genome Informatics, Graduate School of Medicine, The University of Tokyo, Tokyo, Japan; 5 Department of Otorhinolaryngology–Head and Neck Surgery, Graduate School of Medicine, Osaka University, Suita, Japan; 6 Department of Head and Neck Surgery, Aichi Cancer Center Hospital, Nagoya, Japan; 7 Department of Respiratory Medicine and Clinical Immunology, Graduate School of Medicine, Osaka University, Suita, Japan; 8 Laboratory of Immune Regulation, Department of Microbiology and Immunology, Graduate School of Medicine, Osaka University, Suita, Japan; 9 Department of Neurology, Graduate School of Medicine, Osaka University, Suita, Japan; 10 Department of Neurosurgery, Graduate School of Medicine, Osaka University, Suita, Japan; 11 Department of Pediatrics, Graduate School of Medicine, Osaka University, Suita, Japan; 12 Laboratory of Statistical Immunology, WPI Immunology Frontier Research Center (WPI-IFReC), Osaka University, Suita, Japan; 13 Department of Neurology, Suita Municipal Hospital, Suita, Japan; 14 Department of Infection Metagenomics, Research Institute for Microbial Diseases, Osaka University, Suita, Japan; 15 Department of Rheumatology and Allergology, NHO Osaka Minami Medical Center, Kawachinagano, Japan; 16 Center for Infectious Disease Education and Research, Osaka University, Suita, Japan; 17 WPI Immunology Frontier Research Center, Osaka University, Suita, Japan; 18 Department of Immunopathology, Immunology Frontier Research Center, Osaka University, Suita, Japan

**Keywords:** Microbial genetics, Microbial genetics, DNA sequencing

## Abstract

Human DNA present in faecal samples can result in a small number of human reads in gut shotgun metagenomic sequencing data. However, it is presently unclear how much personal information can be reconstructed from such reads, and this has not been quantitatively evaluated. Such a quantitative evaluation is necessary to clarify the ethical concerns related to data sharing and to enable efficient use of human genetic information in stool samples, such as for research and forensics. Here we used genomic approaches to reconstruct personal information from the faecal metagenomes of 343 Japanese individuals with associated human genotype data. Genetic sex could be accurately predicted based on the sequencing depth of sex chromosomes for 97.3% of the samples. Individuals could be re-identified from the matched genotype data based on human reads recovered from the faecal metagenomic data with 93.3% sensitivity using a likelihood score-based method. This method also enabled us to predict the ancestries of 98.3% of the samples. Finally, we performed ultra-deep shotgun metagenomic sequencing of five faecal samples as well as whole-genome sequencing of blood samples. Using genotype-calling approaches, we demonstrated that the genotypes of both common and rare variants could be reconstructed from faecal samples. This included clinically relevant variants. Our approach can be used to quantify personal information contained within gut metagenome data.

## Abstract (web summary)

Personal information such as genetic sex, ancestry and clinically relevant genetic variants can be recovered from human reads present in faecal metagenomic sequencing data.

## Main

Research on the human microbiome, a collection of the microorganisms inhabiting the human body, has been greatly expanded in the past decade. One of the driving forces of this expansion has been the rapid progress of high-throughput metagenome sequencing technologies, including metagenome shotgun sequencing^1^. Metagenome shotgun sequencing, which achieves high taxonomic resolution and applicability to functional profiling^2,3^, has recently been preferred in many situations such as gut microbiome analysis.

In metagenome shotgun sequencing bulk DNA extracted from microbiome samples is directly sequenced without targeted amplification of the bacteria-specific marker genes. Therefore, the non-bacterial component of the samples, including host DNA, are also sequenced. The amount of the host DNA is strongly affected by the sample type, collection method and health status^4,5^. For example, faecal samples typically contain a relatively small amount of host DNA (<10%), whereas DNA extracted from saliva, nasal cavity, skin and vaginal samples is dominated by host-derived components (>90%)^6^. Such host-derived reads are routinely removed from metagenome data before downstream analysis because they could bias the results of the microbial quantification^7–9^.

Host DNA removal is also important to protect the privacy of donors. In the Human Microbiome Project, metagenome data were collected from various body sites, resulting in a notable amount of human DNA^9^. Human reads in the Human Microbiome Project dataset potentially enabled genome-wide variant calling and re-identification of individuals^10,11^. Given that human germline genotype is highly confidential information and care should be taken when shared with the community^12^, human reads in metagenome data should be removed before deposition, especially when personal identifying information should not be accessed. In addition, both the researchers and study participants should recognize that metagenome data include germline genome information.

These previous findings relying on direct germline-variant calling cannot be straightforwardly applied to gut metagenome data. This is because the amount of human DNA in faecal samples is too shallow to perform reliable genotype calling. For example, the mean ratio of the human-derived reads in gut metagenome data was 1% in the Human Microbiome Project^9^ and would result in a human genome coverage of only 0.03× if 10 G base pairs were sequenced. Whether other personal information (that is, sex prediction, re-identification from other datasets and ancestry prediction) can be reconstructed from such a small number of reads has not been quantitatively evaluated and is unclear. To clarify ethical concerns related to data sharing, it is necessary to reveal what kind of personal information could be recovered from gut metagenome data when deposited without human read removal.

Besides ethical concerns, human reads in gut metagenome data can be a useful resource. For example, personal information obtained from general gut metagenome data could be useful for stool-based forensics^13^. In addition, although solid reconstruction of genotypes from gut metagenome data has not been demonstrated, we propose that gut metagenome datasets with a large number of human reads (for example, deep sequencing^14^ or longitudinal collection^15^) can achieve robust variant calling. If successfully reconstructed, such genome-wide genetic information could be used for various applications such as the estimation of disease risks based on polygenic risk scores and pathogenic variants^16,17^. Nevertheless, we should develop optimal methods to reconstruct genotype information even from such deep gut metagenome data but shallow human reads compared with typical human whole-genome sequencing (WGS). Most importantly, we need quantitative discussions on the accuracy of germline-variant calling from metagenome data.

**Fig. 1:**
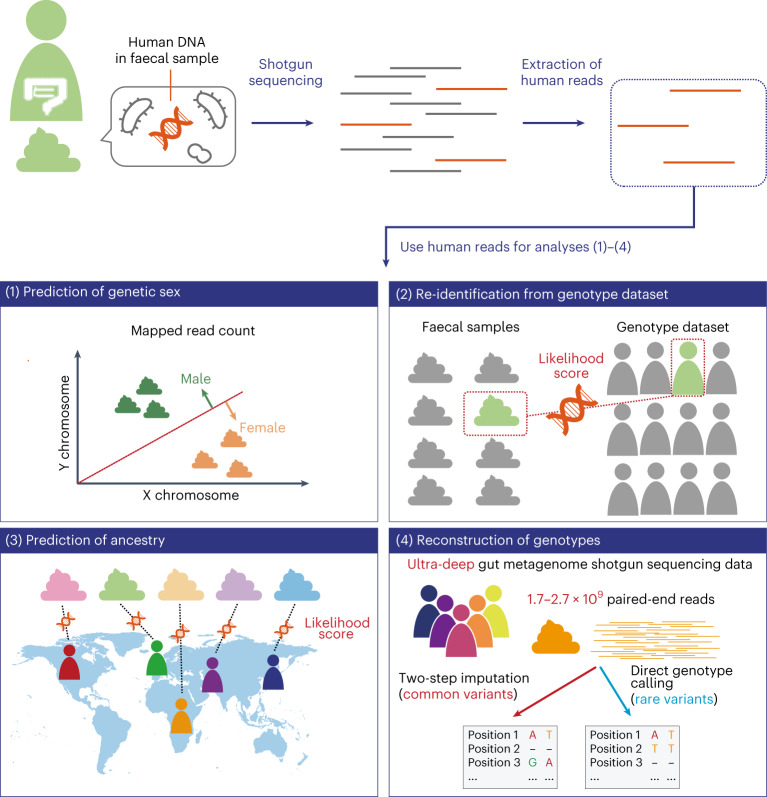
Overview of this study. Schematic overview of the study. We extracted the human reads from the gut metagenome data and then performed the following four analyses using the extracted human reads: (1) prediction of genetic sex, (2) re-identification from the genotype dataset, (3) prediction of ancestry and (4) reconstruction of the genotypes.

Here we developed a series of methods to efficiently reconstruct personal information from a small number of human reads, which we applied to the gut metagenome data of 343 Japanese individuals with available genotype data (Fig. 1). In summary, we: (1) predicted genetic sex based on the sequencing depth of the sex chromosomes; (2) developed a method to re-identify an individual from a genotype dataset based on the human reads in gut metagenome data—our method did not directly call genotype as previously done by Blekhman and colleagues^11^ but rather calculated the likelihood score to efficiently utilize the small number of reads in gut metagenome data; (3) predicted the ancestries of individuals by modifying the likelihood score-based method; and (4) performed ultra-deep gut metagenome shotgun sequencing and reconstructed genotypes by combining two complementary genotype-calling approaches—two-step imputation of common variants and direct calling of rare variants.

## Results

### Extraction of human reads from gut metagenome data

**Fig. 2:**
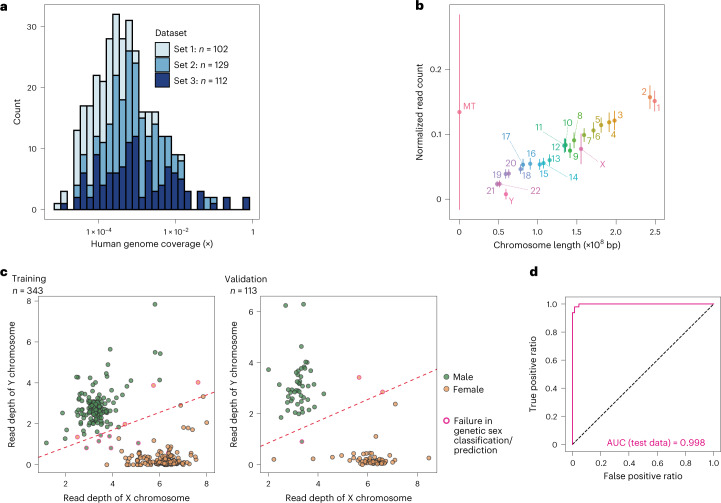
Extraction of human reads from gut metagenome data and genetic sex prediction. **a**, Distribution of human genome coverages of the human reads extracted from the gut metagenome data. The colours of the stacked bars represent the sequencing batch of the gut metagenome data. **b**, Per-chromosome mean normalized read counts of the gut metagenome data. The error bars represent the s.d. Different chromosomes are represented in different colours and labelled with their respective number or character. MT, mitochondrial chromosome. **c**, Normalized read depth of the X and Y chromosomes for the gut metagenome data of the training (left) and validation (right) datasets. Plots were made for the GRCh37 reference genome. The colours of the fills represent the genetic sex of the samples. The colours of the outlines represent whether or not the sex classification (left) or prediction (right) from the human reads extracted from the gut metagenome data is correct. The red dashed lines indicate the thresholds for the Y/X chromosome read-depth ratio determined using logistic regression (Y/X = 0.43). The correct classification or prediction ratios were 96.8% (332/343) and 97.3% (110/113) for the training and validation datasets, respectively. **d**, Receiver operating characteristic curve of the genetic sex prediction for the validation data. AUC, area under the curve. Source data

**Extended Data Fig. 1:**
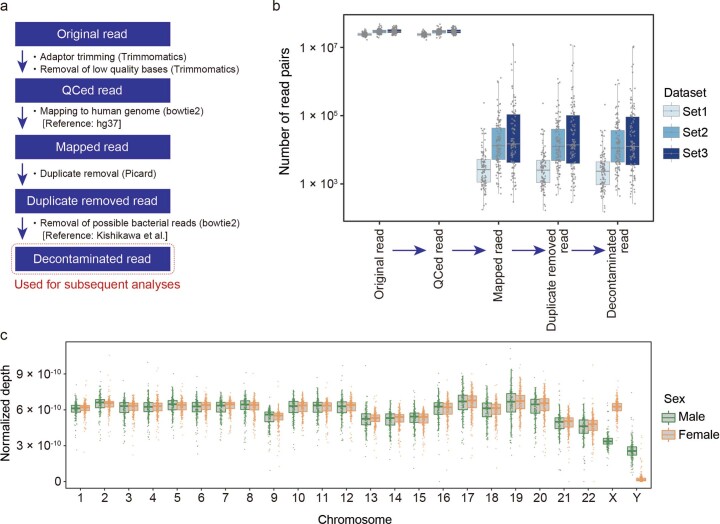
Extraction of human reads from gut metagenome shotgun sequencing data. **a**, Pipeline for the extraction of the human reads from the gut metagenome data. **b**, Number of reads at each process of the human read-extraction pipeline. Boxplots indicate the median values (centre lines) and IQRs (box edges), with the whiskers extending to the most extreme points within the range between (lower quantile − [1.5 × IQR]) and (upper quantile + [1.5 × IQR]). *n* = 102, 129 and 112 biologically independent samples, respectively for datasets 1, 2 and 3. **c**, Normalized depths of the human reads in the gut metagenome data per chromosome. Boxplots indicate the median values (centre lines) and IQRs (box edges), with the whiskers extending to the most extreme points within the range between (lower quantile − [1.5 × IQR]) and (upper quantile + [1.5 × IQR]). *n* = 147 and 196 biologically independent samples, respectively, for male and female individuals. IQR, interquartile ranges; QC, quality control.

We first constructed a pipeline to extract human reads from gut metagenome data (Extended Data Fig. 1a). We applied this pipeline to the gut metagenome data, composed of the three sequencing batches, of 343 Japanese individuals (Supplementary Table 1). The average number of reads in the original, human read and decontaminated-human read data were 2.8 × 10^7^, 1.4 × 10^5^ (0.54% of the original reads) and 1.2 × 10^5^ (0.46% of the original reads), respectively (Extended Data Fig. 1b and Supplementary Table 2). The human genome coverages of the extracted human reads ranged from 0.000010× to 0.78× and varied greatly across samples (Fig. 2a and Supplementary Table 2; mean, 0.0088×; s.d., 0.060×; median, 0.00044×). The normalized human read counts per chromosome were proportional to the chromosome length for autosomes (Fig. 2b), suggesting that the human reads in the gut metagenome data were derived uniformly from every chromosome. Apparent differences in the read depths between sexes were not observed for the autosomes (Extended Data Fig. 1c).

In our evaluation of the read quality human-derived reads tended to have a greater read-through ratio and shorter length after quality control than other reads (Supplementary Fig. 1a–d and Supplementary Note 1). Human mitochondrial DNA (mtDNA)-based analyses suggested possible contamination of non-host human reads in a handful of cases (Supplementary Fig. 2a–e and Supplementary Note 1).

### Genetic sex prediction based on gut metagenome data

Motivated by the fact that the human reads in the gut metagenome data were derived uniformly from every chromosome, we predicted the genetic sex of the samples based on the read depths of the non-pseudo-autosomal regions of the sex chromosomes. As expected, the read depth of the X chromosome was almost twice higher in females than males (Fig. 2c). The read depth of the Y chromosome was much higher in male samples than female samples.

In a logistic regression analysis, a Y-to-X chromosome read-depth ratio of 0.43 was the best threshold for classifying the genetic sex of the gut metagenome data of the 343 individuals (Fig. 2c). We applied this threshold to the validation dataset (*n* = 113) and the genetic sex of 97.3% of the samples was correctly predicted (Fig. 2c,d and Supplementary Table 3). The number of sex chromosomal reads tended to be small in the handful of cases where the genetic sex classification and prediction failed; other factors such as contamination of non-host human DNA might also contribute to the misclassification and misprediction (Supplementary Fig. 3). The difference in the Y-to-X chromosome read-depth ratio between sexes did not depend on the method used to extract the stool DNA (phenol–chloroform extraction versus DNeasy PowerSoil kit-based extraction; Supplementary Fig. 4a,b).

**Extended Data Fig. 2:**
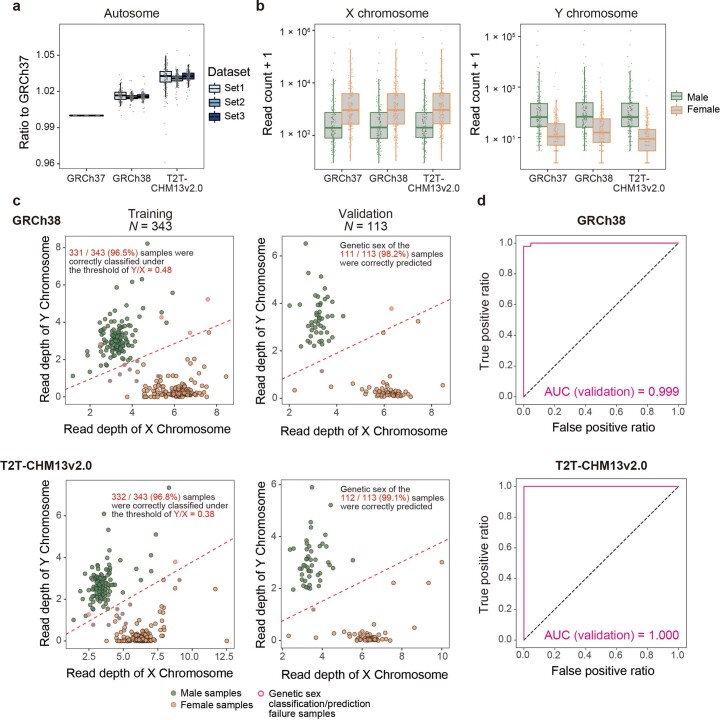
Genetic sex prediction based on the human reference genome GRCh38 and T2T-CHM13v2.0. **a**, Boxplots indicate the relative number of the autosomal reads defined based on each reference genome. Ratios to the GRCh37 are indicated. *n* = 102, 129 and 112 biologically independent samples, respectively, for datasets 1, 2 and 3. **b**, Boxplots indicate the number of the sex chromosomal reads defined based on each reference genome. *n* = 147 and 196 biologically independent samples, respectively, for male and female individuals. **c**, Scatter plots depicting normalized read depths of the X chromosome (*x* axis) and Y chromosome (*y* axis) for the gut metagenome data of the training (left) and validation (right) datasets. Plots for different reference genomes (top: GRCh38; bottom: T2T-CHM13v2.0). The colours of the fills represent the genetic sex of the samples. The colours of the outlines represent whether or not the sex classification (left) or prediction (right) from the human reads extracted from the metagenome data is correct. The red dashed lines indicate the thresholds for the Y/X chromosome read-depth ratio determined by logistic regression. **d**, Receiver operating characteristic curve of the genetic sex prediction for the validation data (top: GRCh38; bottom: T2T-CHM13v2.0). Boxplots indicate the median values (centre lines) and IQRs (box edges), with the whiskers extending to the most extreme points within the range between (lower quantile − [1.5 × IQR]) and (upper quantile + [1.5 × IQR]). AUC, area under the curve; IQR, interquartile ranges.

Analyses with different human reference genomes (GRCh38 and the telomere-to-telomere reference genome chm13v2.0 (T2T-CHM13v2.0) (ref. ^18^) suggested a potential superiority of the sex prediction performance in the T2T-CHM13v2.0 but the difference between the reference genomes was too small to draw a definitive conclusion (Extended Data Fig. 2a–d, Supplementary Table 3 and Supplementary Note 2).

### Overview of the re-identification analysis

**Fig. 3:**
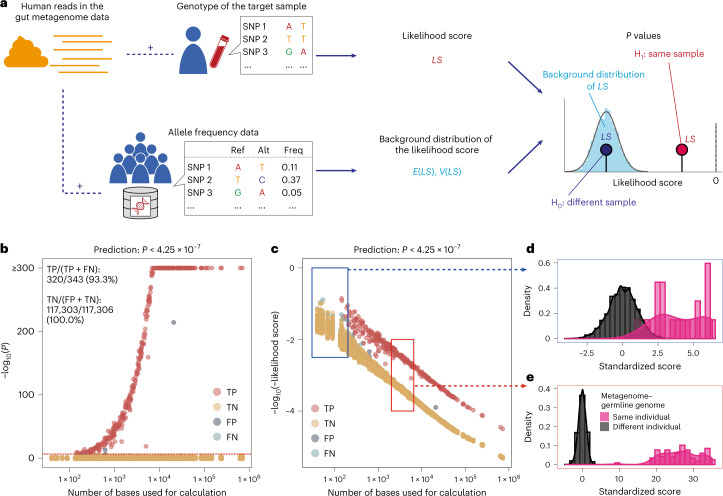
The likelihood score-based method identified the gut metagenome and genotype data pairs derived from the same individuals. **a**, Schematic of the likelihood score-based identification of the gut metagenome shotgun sequencing and genotype data pairs derived from the same individuals. Human reads were extracted from the gut metagenome data, and the likelihood score was calculated using the human reads and individual genotype data. We also calculated the expected value and variance of the likelihood score from the human reads and reference allele frequency data. Under the following null (H_0_) and alternative (H_1_) hypotheses, we calculated the *P* values for the gut metagenome data–genotype data pairs: H_0_, gut metagenome and genotype data were derived from the same individual; H_1_, gut metagenome and genotype data were derived from different individuals. **b**,**c**, Re-identification from a set of genotype data based on the human reads in the gut metagenome data of 343 individuals. *P* values were calculated using a one-sided test for the likelihood score as indicated in **a** (Methods). We applied multiple-test correction to the significance threshold (*P* = 0.05; 117,649 tests). *P* values (**b**) and likelihood scores (**c**) versus the number of bases used for calculation. The results of the 117,649 tests (343 genotype data × 343 metagenome data) are indicated as the colours of the points. **d**,**e**, The distribution of the standardized likelihood score is indicated for the regions indicated with blue (**d**) and red (**e**) boxes in **c** stratified according to whether or not the derivation of the gut metagenome data and genotype data are the same. Alt, alternative allele; freq, frequency; ref, reference allele; FN, false negative; FP, false positive; TN, true negative; and TP, true positive. Source data

Re-identification from biobank data has attracted ethical concerns mainly in the context of the deposition of an individual’s genotype information for >30 independent single nucleotide polymorphism (SNP) sites or a relatively large number of the human read data^12^. Given that definitive genotype calling from the small number of human reads was technically challenging, whether we could re-identify individuals with gut metagenome data from a set of genotype data had not been quantitatively evaluated. To evaluate this, we developed a likelihood score-based method to efficiently utilize the information from genomic regions with low coverage (Fig. 3a).

The likelihood score was calculated from the human reads in the metagenome data and the genotype data of the target sample. We extracted base information on every human read that covered known SNP sites and calculated the likelihood score. The likelihood score reflected the probability that the observed human reads in the gut metagenome data were derived from the target genotype data. We also calculated the expected values and variances of the likelihood scores from the population allele frequency data and plotted the *P* values, which indicated whether the likelihood score was significantly higher than that obtained from the unrelated metagenome data–genotype data pair. This method was suitable for the gut metagenome data because we could utilize information from the genomic regions with low coverage.

### Simulation analysis of the re-identification

**Extended Data Fig. 3:**
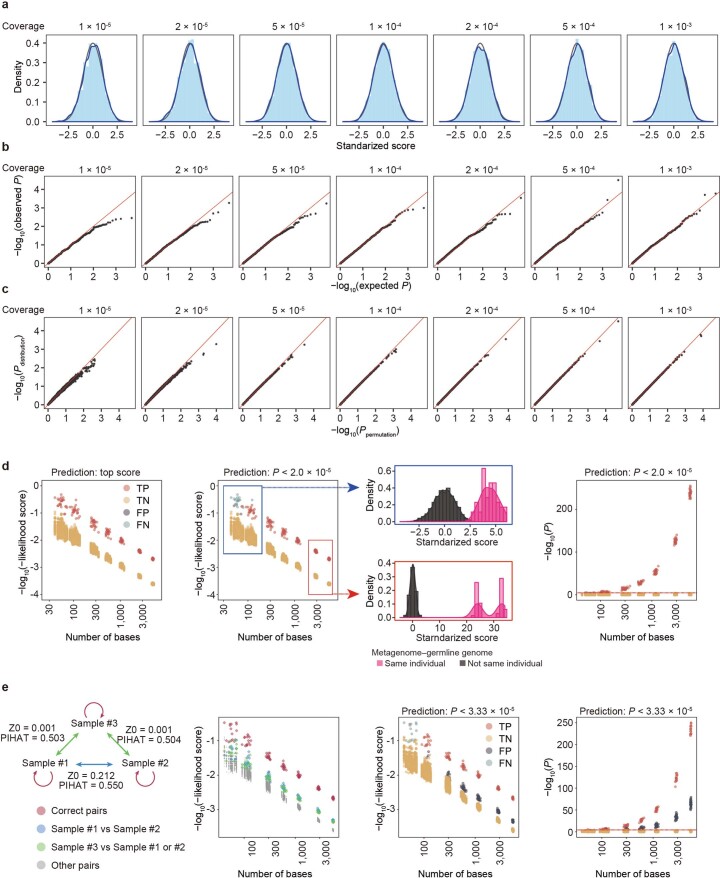
Simulation experiment for the identification of pairs of read and genotype data derived from the same individuals. **a**, Histograms represent the distribution of the standardized likelihood score for which the read data and genotype data originate from different samples. The blue line indicates the density plot made from the simulation results and the black line indicates the normal distribution. **b**, Q-Q plots representing the relationship between the observed and expected *P* values in the cases where the read data and genotype data originate from different samples. The red line indicates *y* = *x*. **c**, Scatter plots represent the relationship between the *P* values calculated from the background distribution of the score (*P*_distribution_) and *P* values empirically calculated with the permutation procedure (*P*_permutation_) for the cases where the read and genotype data originate from different samples. The red line indicates *y* = *x*. **d**, Re-identification from a set of genotype data based on the 175 (5 WGS data × 5 random seeds × 7 coverages) simulated read data. The *x* axis of the scatter plots indicates the number of the bases used for the calculation of the likelihood scores. The *y* axis of the scatter plots indicates the likelihood scores (left and middle) or *P* values (right). The results of the 17,500 tests (100 genotype data × 25 simulated data × 7 coverages) are indicated as the colour of the points. The identification of the pairs of the read and genotype data is based on the top score (left) or *P* values (middle and right). The distribution of the standardized likelihood score is indicated for a part of the middle plot with the stratification by whether the derivation of the simulated read data and the genotype data are the same or not. **e**, Re-identification from a set of genotype data based on the 105 (3 WGS data × 5 random seeds × 7 coverages) simulated read data. The three samples with the WGS data have a familial relationship as indicated. The *x* axis of the scatter plots indicates the number of the bases used for the calculation of the likelihood scores. The *y* axis of the scatter plots indicates the likelihood scores (left and middle) or *P* values (right). The colours of the points represent the categories of the pairs of the read data and the genotype data (left) or the result of the *P* value-based predictions (100 genotype data × 15 simulated data × 7 coverages = 10,500 tests). FN, false negative; FP, false positive; TN, true negative; TP, true positive.

**Extended Data Fig. 4:**
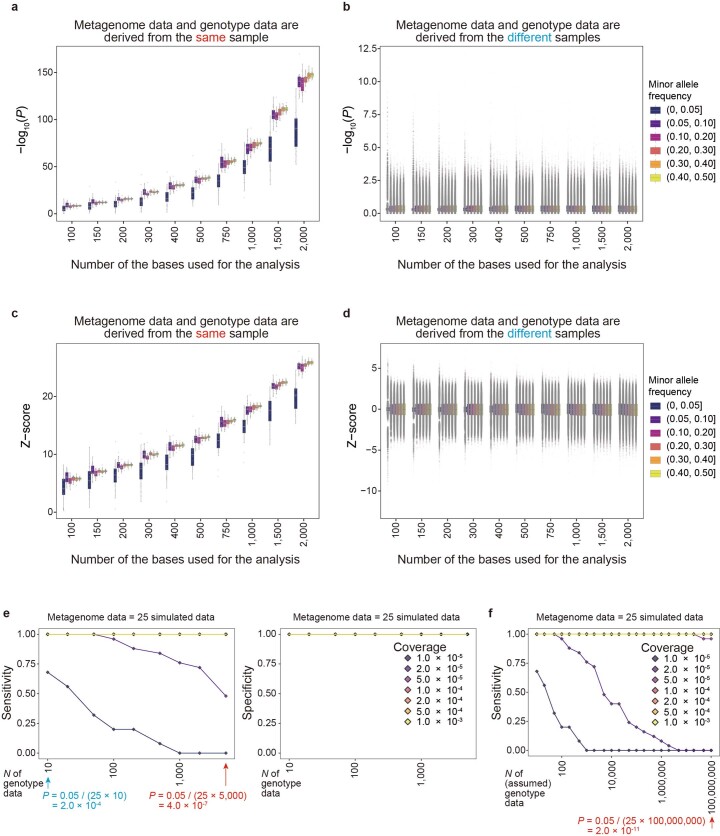
Effects of the allele frequency and number of the genotype data on the efficiency of the re-identification. **a**–**d**, Boxplots indicate the *P* values (**a**,**b**) and *Z*-scores (**c**,**d**) in the re-identification analysis with different numbers and MAFs of the SNPs. Cases where the metagenome data and genotype data are derived from the same sample (**a**,**c**; 5 samples × 5 seeds × 1 genotype data) and different samples (**b**,**d**; 5 samples × 5 seeds × 99 genotype data) are indicated. Boxplots indicate the median values (centre lines) and IQRs (box edges), with the whiskers extending to the most extreme points within the range between (lower quantile − [1.5 × IQR]) and (upper quantile + [1.5 × IQR]). **e**, Re-identification from different sizes of genotype data (10–5,000) based on the simulated read data. The *y* axis indicates the sensitivity (left) and specificity (right). The *x* axis indicates the size of the genotype data. The colours of the dots indicate the human genome coverages of the simulated read data. **f**, Re-identification under the different significance thresholds based on the simulated read data. The *y* axis indicates the sensitivity and the *x* axis indicates the assumed size of the genotype data. The colours of the dots indicate the human genome coverages of the simulated read data. IQR, interquartile ranges; SNP, single nucleotide polymorphism.

We evaluated the distribution of the likelihood scores for the simulated human reads (downsampled WGS data) with a range of human genome coverages (1 × 10^−5^× to 1 × 10^−3^×). The standardized likelihood scores were almost normally distributed when the read and genotype data were derived from different individuals (Extended Data Fig. 3a). *P* values calculated from the standardized likelihood scores were not inflated compared with the expected and permutation-based *P* values (Extended Data Fig. 3b,c). In the simulation experiment genotype data with the highest scores were always derived from the same individual that the read data obtained (Extended Data Fig. 3d). The read data–genotype data pairs derived from the same individuals were predicted based on the *P* values. Although the re-identification worked well with human genome coverages ≥0.00005×, false negatives were observed when the human genome coverages were ≤0.00002× (20% and 96% sensitivity, respectively, for 0.00001× and 0.00002× coverage; Extended Data Fig. 3d and Supplementary Table 4). This was due to insufficient deviation of the non-null distribution of the likelihood score from the null distribution. Although the genotype data with the highest scores correctly pinpointed from whom the read data came, prediction based on the *P* values caused false positives due to familial relationships (Extended Data Fig. 3e). The power of the analysis with only the relatively rare SNP sites (minor allele frequency (MAF) < 5%) was slightly lower than the analyses with more common SNP sites (Extended Data Fig. 4a–d and Supplementary Note 3). The re-identification method had enough power even in the simulation analyses with large sample sizes or non-host human DNA contamination (Extended Data Fig. 4e,f, Supplementary Fig. 5a–f and Supplementary Note 3).

### Re-identification based on gut metagenome data

**Extended Data Fig. 5:**
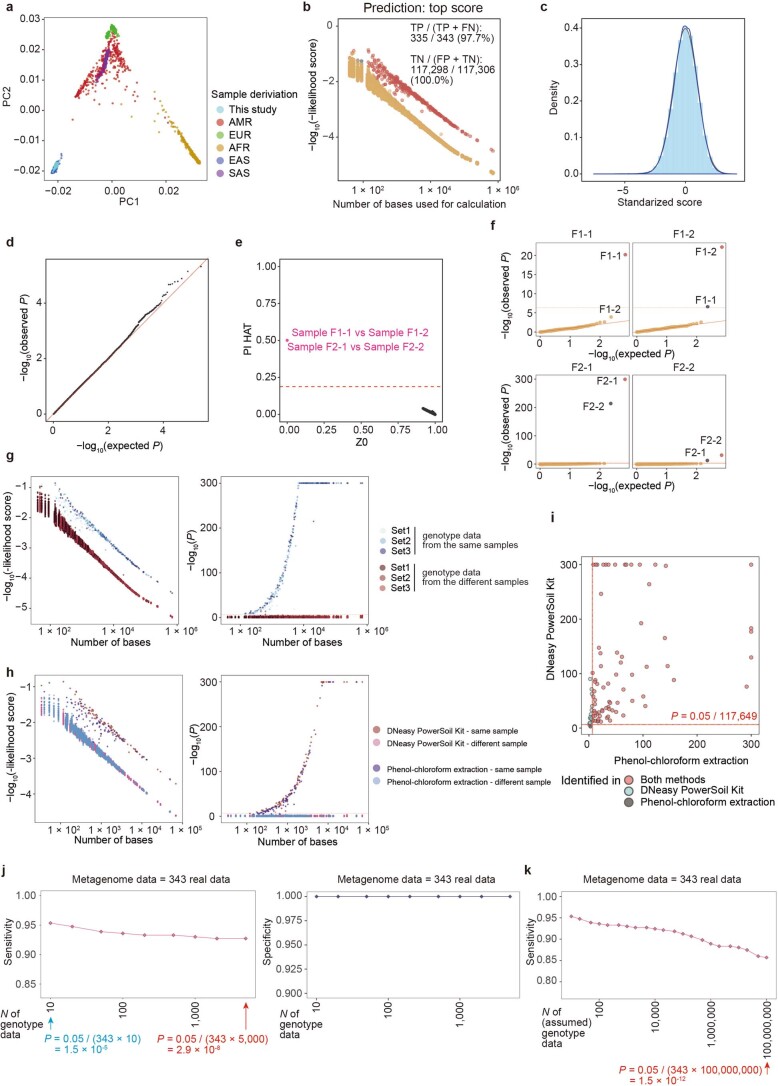
Re-identification from a set of genotype data based on the human reads in the gut metagenome shotgun sequencing data. **a**, Results of the PCA analysis of the 343 in-house genotype data and the 1KG data. **b**, Re-identification from a set of genotype data based on the human reads in the 343 gut metagenome data. The *x* axis of the plots indicates the number of bases used for the calculation of the likelihood scores. The *y* axis of the plot indicates the likelihood scores. The genotype data with the highest likelihood score are predicted to sharing the same derivation with the gut metagenome data. The results of the 117,649 tests (343 genotype data × 343 gut metagenome data) are indicated as the colour of the points. **c**, Histograms represent the distribution of the standardized likelihood score for which the gut metagenome data and the genotype data originate from unrelated samples. The blue line indicates the density plot made from the simulation results and the black line indicates the normal distribution. **d**, Q-Q plot representing the relationship between the observed and expected *P* values in the cases where the gut metagenome and genotype data originate from unrelated samples. The red line indicates *y* = *x*. **e**, Scatter plots indicating kinship between samples. The *x* axis indicates Z0 (ratio of the SNPs with the identity by state = 0) and the *y* axis indicates PIHAT. The suggested kinships are coloured pink. **f**, Q-Q plots representing the results of the likelihood score-based tests for the samples from the two putative families suggested in **e**. The red horizontal line represents *P* = 4.25 × 10^−7^. **g**, Re-identification from a set of genotype data based on the human reads in the 343 gut metagenome data. The *x* axis of the plots indicates the number of bases used for the calculation of the likelihood scores. The *y* axis of the plot indicates the likelihood scores (left) or *P* values (right). For the results of the 117,649 tests (343 genotype data × 343 metagenome data), (i) datasets of the metagenome data and (ii) whether the metagenome data and genotype data are derived from the same individual are indicated as the colours of the points. **h**, Scatter plot representing the relationship between likelihood scores (left) or *P* values (right; y axis) and the number of bases used for the re-identification (*x* axis). Results for the metagenome data obtained from the same 88 samples (a subset of samples in the dataset 1) through the DNA extraction method used in the main analyses (phenol–chloroform extraction) and kit-based DNA extraction (DNeasy PowerSoil Kit) are indicated as different dots with different colours (88 metagenome data × 88 genotype data × 2 methods = 15,488 dots). Whether the metagenome data and genotype data are obtained from the same individuals or not are also indicated as colours of the dots. **i**, Scatter plot representing the *P* values obtained by the re-identification methods. Results for the metagenome data obtained from the same 88 samples through the DNA extraction method used in the main analyses (phenol–chloroform extraction, *x* axis) and kit-based DNA extraction (DNeasy PowerSoil Kit, *y* axis) are indicated. Only results for the genotype data obtained from the same individuals as the metagenome data are indicated. Both red dashed lines represent *P* = 0.05 / 117,649. The colours of the dots indicate whether or not the re-identification succeeds under the significance thresholds of *P* < 0.05 / 117,649. **j**, Re-identification from different sizes of genotype data (10–5,000) based on the human reads in the 343 gut metagenome data. The *y* axis indicates the sensitivity (left) and specificity (right). The *x* axis indicates the size of the genotype data. **k**, Re-identification under the different significance thresholds based on the human reads in the 343 gut metagenome data. The *y* axis indicates the sensitivity and the *x* axis indicates the assumed size of the genotype data. 1KG, 1000 Genome Project; AFR, African; AMR, American; EAS, East Asian; EUR, European; FN, false negative; FP, false positive; PC, principal components; SAS, South Asian; TN, true negative; TP, true positive.

We performed an experiment with the gut metagenome data of 343 Japanese individuals and imputed genotype data obtained with the SNP array (Extended Data Fig. 5a). We predicted gut metagenome data–genotype data pairs derived from the same individuals based on the *P* values. We achieved 93.3% (320/343) sensitivity and 100.0% (117,303/117,306) specificity (Fig. 3b and Supplementary Table 5). Samples with >100 bases (minimum number of the reads, 3.8 × 10^2^) had 98.2% (332/338) sensitivity and samples with >1,000 bases (minimum number of the reads, 2.7 × 10^3^) had 98.7% (229/232) sensitivity. Therefore, a portion of the false negatives were caused by an insufficient number of bases due to insufficient deviation of the non-null distribution of the likelihood score from the null distribution (Fig. 3c–e). The genotype data with the highest scores did not have the same derivation as the gut metagenome data in 2.3% (8/343) of cases; this might be due to several factors (that is, too few bases and contamination of non-host human reads; Extended Data Fig. 5b). The null standardized likelihood scores were distributed normally and the null *P* values were not inflated in the real data analysis (Extended Data Fig. 5c,d). As suggested from the simulation experiment, familial relationships contributed to all three false positives detected in the prediction based on the *P* values (Extended Data Fig. 5e,f). The relationship between the number of bases, likelihood scores and *P* values was consistent across the three metagenome datasets (Extended Data Fig. 5g). The relationship between the number of the bases, likelihood scores and *P* values was unaffected by the DNA extraction method used (Extended Data Fig. 5h,i). Therefore, our likelihood score-based method could re-identify individuals from a set of genotype data based on the human reads in the gut metagenome data.

To evaluate the effect of sample size, we performed analyses with a wide range of genotype data sizes (10–5,000 unrelated individuals) or significance thresholds (*P* = 1.5 × 10^−5^–1.5 × 10^−12^). The type 1 error was controlled (Extended Data Fig. 5j) and the matched genotype data of 294/343 (85.7%) gut metagenome data were correctly identified under the strict significance threshold (*P* = 1.5 × 10^−12^, corresponding to the 343 metagenome data × 100,000,000 genotype data; Extended Data Fig. 5k). These findings suggested the risk of re-identification in a case where metagenome shotgun sequencing-based research participants simultaneously took part in a different biobank-scale genome study.

**Extended Data Fig. 6:**
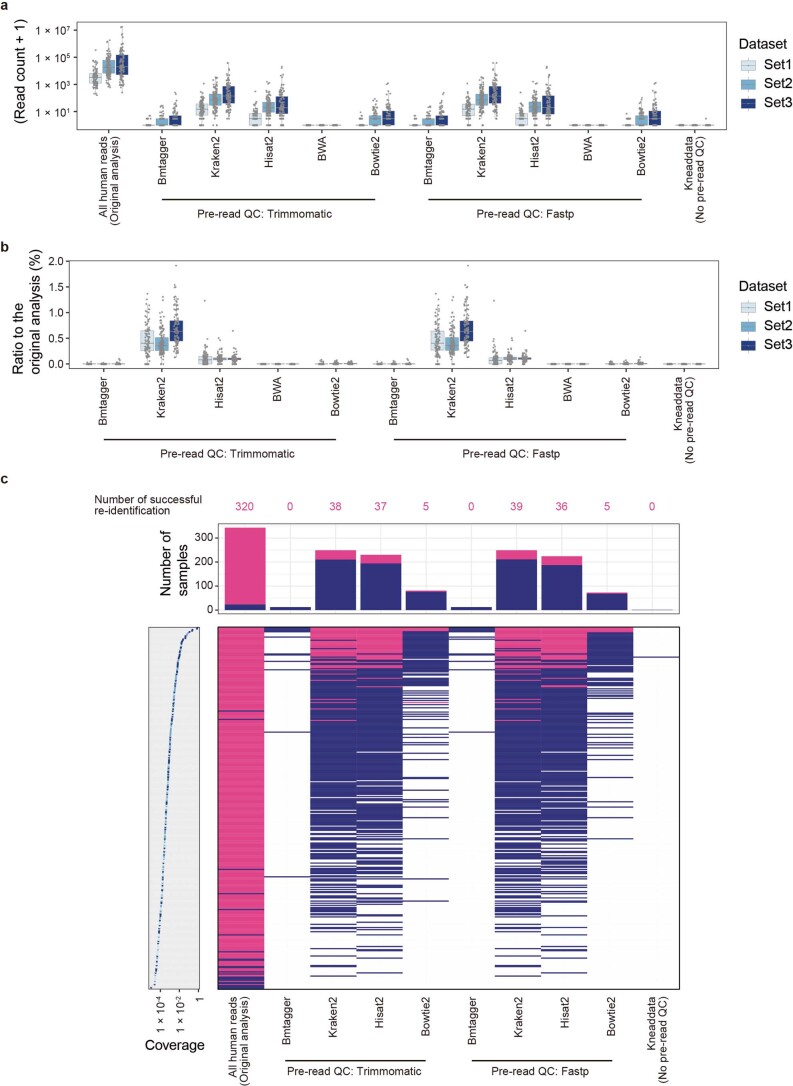
Re-identification after human read filtering. **a**, Number of human reads in the gut metagenome data after human read filtering per dataset. Boxplots indicate the median values (centre lines) and IQRs (box edges), with the whiskers extending to the most extreme points within the range between (lower quantile − [1.5 × IQR]) and (upper quantile + [1.5 × IQR]). *n* = 102, 129 and 112 biologically independent samples, respectively, for datasets 1, 2 and 3. **b**, The ratio of the human reads in the gut metagenome data remained even when the human read filtering was applied. Per-datasets boxplots indicate the median values (centre lines) and IQRs (box edges), with the whiskers extending to the most extreme points within the range between (lower quantile − [1.5 × IQR]) and (upper quantile + [1.5 × IQR]). *n* = 102, 129 and 112 biologically independent samples, respectively for datasets 1, 2 and 3. **c**, A tile plot represents the results of the re-identification after human read filtering. The magenta indicates the success of re-identification (*P* < 0.05 / 117,649) and the navy indicates the failure of re-identification. No fill means that there are no bases that can be used for the analysis and re-identification is not applicable. Only the human read filtering methods, in which at least a sample was used for the re-identification analysis, are indicated. The left panel indicates the human genome coverages of the metagenome data. The bar plot on the top indicates the number of successful (magenta) and failed (navy) re-identification. QC, quality control.

We performed re-identification analysis after applying several kinds of human read filtering methods and evaluated whether it was sufficient to prevent re-identification. Although all the human read filtering methods efficiently reduced the number of extracted human reads, filtering with Kraken 2 or HISAT2 sometimes left >1,000 human reads (Extended Data Fig. 6a,b). When the original human genome coverage was high (>1.0 × 10^−2^×), re-identification sometimes worked even after human read filtering using Bowtie 2 (Extended Data Fig. 6c). Therefore, researchers and study participants should recognize that identifiable information could be left in the gut metagenome data even after human read filtering in some exceptional cases.

### Ancestry prediction based on gut metagenome data

**Fig. 4:**
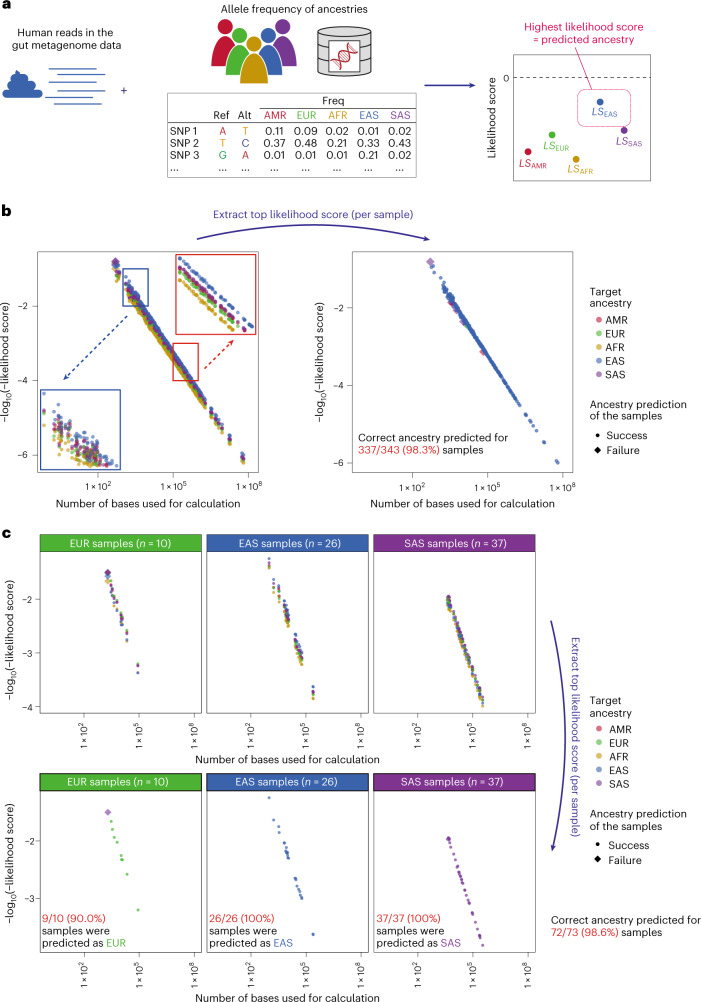
The likelihood score-based method successfully predicted the ancestries of the gut metagenome data. **a**, Schematic of the likelihood score-based method used to predict the ancestry of individuals. Human reads were extracted from the gut metagenome data, and the likelihood score was calculated using the human reads and allele frequency data of the five ancestries calculated from the 1KG dataset. The ancestry with the top likelihood score was predicted as the ancestry of the target sample. **b**, Prediction of the ancestries of the 343 gut metagenome data. The 1,715 scores (5 target ancestries × 343 metagenome data; left) as well as the top scores for each sample are depicted (right). Insets: magnified views of the regions in the coloured boxes. **c**, Prediction of the ancestries of the gut metagenome data of the 73 individuals in our in-house multi-ancestry dataset. The 365 scores (5 target ancestries × 73 metagenome data; top) as well as the top scores for each sample (bottom) are depicted. Plots were generated separately according to the self-reported ancestries of the samples. Alt, alternative allele; freq, frequency; and ref, reference allele. Source data

Whether the ancestry of individuals can be predicted from their gut metagenome data is also a point that needs to be addressed given that genetic ancestry is private information that can reveal the ancestral roots as well as disease risks of an individual^19^. Although genetic ancestry had been inferred from genome-wide variant information^20^, such methods were not applicable to gut metagenome data with very low human genome coverages. Therefore, we modified the likelihood score-based methods for genetic ancestry prediction to efficiently utilize the information from the genomic regions with low coverage (Fig. 4a). We calculated the likelihood score that reflected the likelihood that the observed human reads in the gut metagenome data were obtained from a specified ancestry. In this study we used 1000 Genomes Project (1KG) data as a reference and calculated the likelihood scores for five ancestries, namely American (AMR; admixture of European, African and Native American^20^), European (EUR), African (AFR), East Asian (EAS) and South Asian (SAS). The ancestry with the highest likelihood score among the five ancestries would be a prediction.

**Extended Data Fig. 7:**
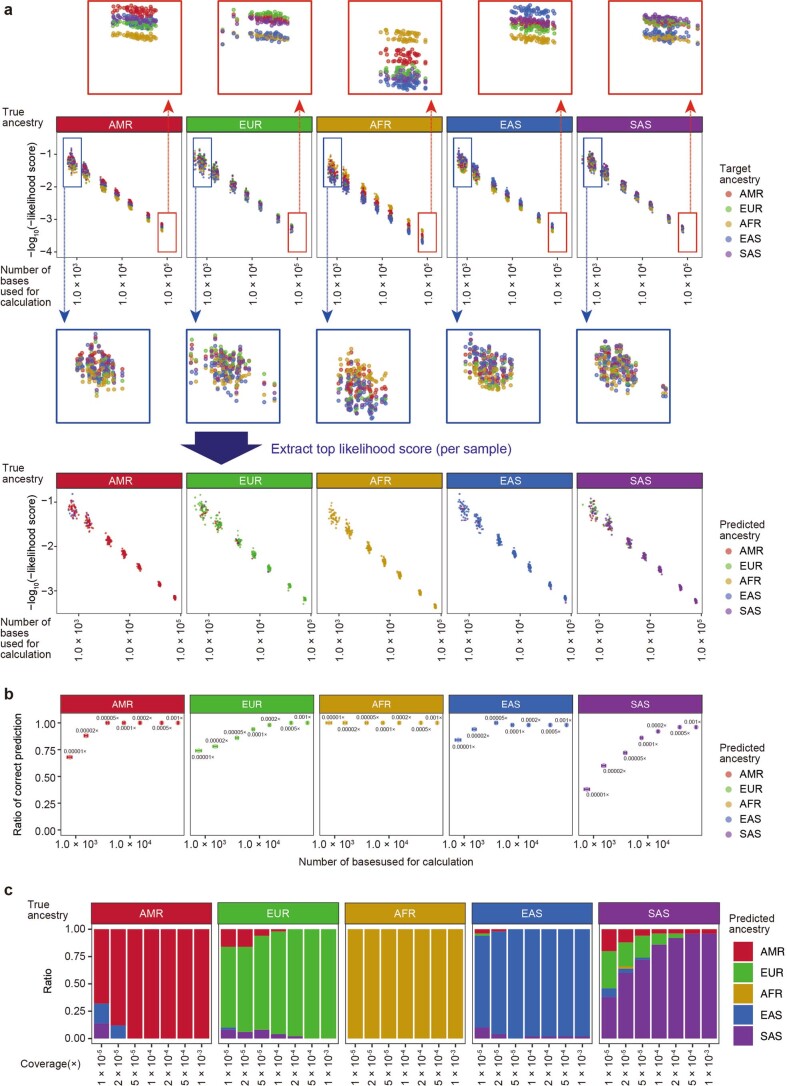
Simulation experiment of the prediction of the ancestry from the small number of the sequencing reads. **a**, Prediction of the ancestries of the simulated 1,750 (50 samples × 5 ancestries × 7 coverages) read data. The *x* axis of the plots indicates the number of bases used for the calculation of the likelihood scores. The *y* axis of the plot indicates the likelihood score. The 8,750 scores (5 target ancestries × 1,750 read data) are indicated in the top plot and the top scores for each read data were depicted in the bottom plot. The colours of the points indicate the target ancestries and the shapes of the points indicate whether or not the ancestry prediction is correct. **b**, Plots representing the correct prediction ratio for each human genome coverage. The *y* axis of the plot indicates the correct prediction ratio. The *x* axis of the plots indicates the number of bases used for the calculation of the likelihood scores. The error bars indicate the s.d. The numbers attached to the points indicate the human genome coverage. **c**, Bar plots representing the composition of the predicted ancestries at each human genome coverage. The colours of the bar represent the predicted ancestries. AFR, African; AMR, American; EAS, East Asian; EUR, European; SAS, South Asian.

In the simulation analysis 0.001× human genome coverage was sufficient to achieve a 0.99 correct prediction ratio (Supplementary Tables 6,7, Extended Data Fig. 7a,b and Supplementary Note 4). When the number of bases was small, ancestry prediction was relatively difficult, especially for SAS individuals, possibly due to the genetic diversity of the SAS population^20^ not being reflected in the 1KG dataset (Extended Data Fig. 7c, Supplementary Fig. 6 and Supplementary Note 4). The prediction accuracy was relatively low when we used only the relatively rare SNP sites (MAF < 5%; Supplementary Fig. 7).

**Extended Data Fig. 8:**
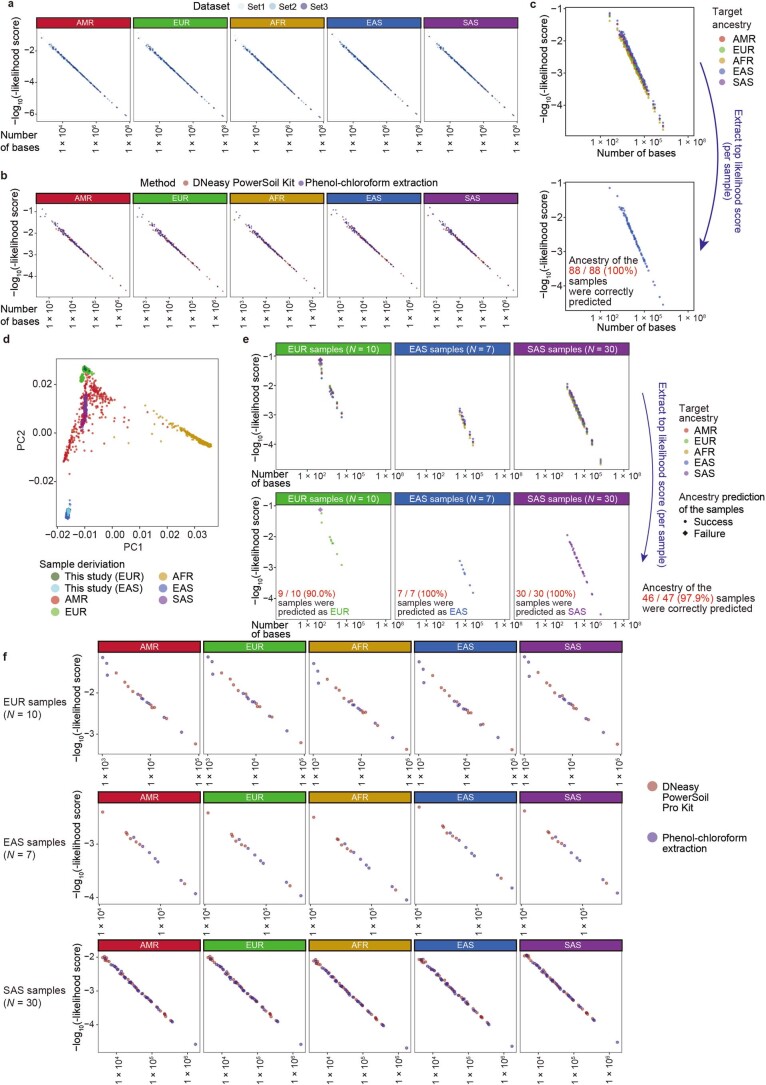
Effects of sequencing batches, DNA extraction method and sample ancestry on the performance of the ancestry prediction. **a**, Scatter plot representing the relationship between the likelihood scores (*y* axis) and the number of bases used for the re-identification (*x* axis) for the five populations. Results for the 343 metagenome data obtained from the three different datasets are indicated as different dots with different colours. **b**, Scatter plot representing the relationship between the likelihood scores (*y* axis) and the number of bases used for the re-identification (*x* axis) for the five populations. Results for the metagenome data obtained from the same 88 samples (a subset of samples in dataset 1) through the DNA extraction method used in the main analyses (phenol–chloroform extraction) and kit-based DNA extraction (DNeasy PowerSoil Kit) are indicated as different dots with different colours. **c**, Prediction of the ancestries of the gut metagenome data of 88 samples which were subjected to the kit-based DNA extraction. The *x* axis of the plots indicates the number of bases used for the calculation of the likelihood scores. The *y* axis of the plot indicates the likelihood score. All scores are indicated in the top plot and the top scores for each sample are depicted in the bottom plot. The colours of the points indicate the target ancestries. **d**, Results of the PCA analysis of the 36 in-house multi-ancestry genotype data (*N*_EUR_ = 10 and *N*_EAS_ = 26) and the 1KG data. **e**, Prediction of the ancestries of the 47 in-house multi-ancestry gut metagenome data that were based on the kit-based DNA extraction with DNeasy PowerSoil Pro Kit (a subset of samples in the in-house multi-ancestry dataset). The *x* axis of the plots indicates the number of bases used for the calculation of the likelihood scores. The *y* axis of the plot indicates the likelihood score. The 235 scores (5 target ancestries × 47 metagenome data) are indicated in the top plot and the top scores for each sample are depicted in the bottom plot. The colours of the points indicate the target ancestries and the shapes of the points indicate whether or not the ancestry prediction is correct. Plots were generated separately according to the self-reported ancestries of the samples (left, EUR; middle, EAS; right, SAS). **f**, Scatter plot representing the relationship between the likelihood scores (*y* axis) and the number of bases used for the re-identification (*x* axis) for the five populations. Results for the metagenome data obtained from the same 47 samples in the in-house multi-ancestry dataset through the two different DNA extraction (phenol–chloroform extraction or DNeasy PowerSoil Pro Kit) are indicated as different dots with different colours. AFR, African; AMR, American; EAS, East Asian; EUR, European; SAS, South Asian.

We predicted the ancestries of the dataset of the 343 gut metagenomes, all of which were EAS samples. We successfully predicted 98.3% (337/343) of the samples as EAS-derived (Fig. 4b and Supplementary Table 8). The misclassification of a portion of the remaining samples could be attributed to low numbers of available bases (≤5.7 × 10^3^ bases; Supplementary Fig. 8). Other factors, such as contamination of non-host human reads, probably contributed to the misclassification of samples with relatively large numbers of available bases (≥1.4 × 10^4^ bases; Supplementary Fig. 8). The relationship between the number of bases and likelihood scores was consistent across the datasets (Extended Data Fig. 8a). The DNA extraction method did not affect the relationship between the number of bases and the likelihood scores (Extended Data Fig. 8b,c).

**Extended Data Fig. 9:**
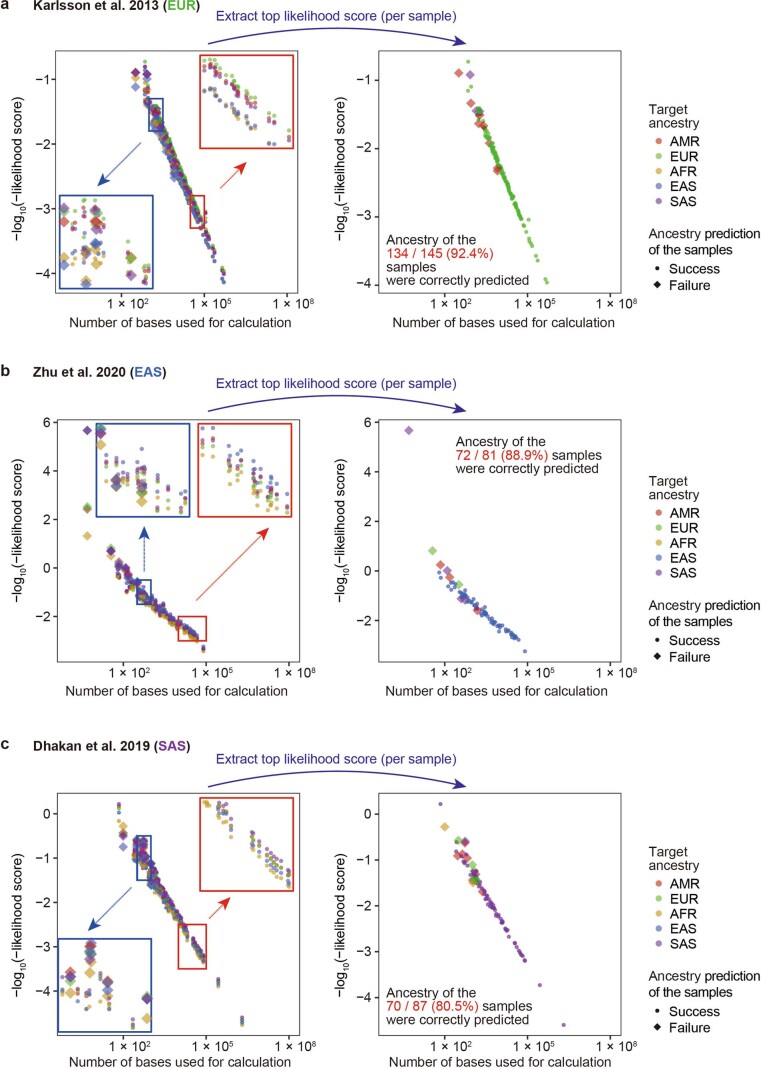
Results of the prediction of the ancestry from the publicly available gut metagenome shotgun sequencing datasets. **a**–**c**, Prediction of the ancestries of the gut metagenome data of EUR (**a**), EAS (**b**) and SAS (**c**) ancestries. The *x* axis of the plots indicates the number of bases used for the calculation of the likelihood scores. The *y* axis of the plot indicates the likelihood score. All scores are indicated in the left plot and the top scores for each sample are depicted in the right plot. The colours of the points indicate the target ancestries and the shapes of the points indicate whether or not the ancestry prediction is correct. We observed regional differences in the correct prediction ratios for the SAS dataset (Dhakan et al., 73.5% (36/49) and 89.5% (34/38) in Bhopal, India and Kerala, India, respectively), which might be also due to the complex genetic features of SAS. AFR, African; AMR, American; EAS, East Asian; EUR, European; SAS, South Asian.

To further evaluate the performance of the ancestry prediction based on gut metagenome data, we performed the analyses with an in-house multiple-ancestry dataset (*n*_EUR_ = 10, *n*_EAS_ = 26 and *n*_SAS_ = 37). We achieved a 98.6% true prediction ratio and a high true prediction ratio was consistently observed across the different ancestries and DNA extraction methods (Fig. 4c, Extended Data Fig. 8d–f and Supplementary Table 8). We also analysed publicly available gut metagenome datasets, namely datasets from Karlsson et al.^21^ (EUR), Zhu et al.^22^ (EAS) and Dhakan et al.^23^ (SAS), and achieved 92.4%, 88.9% and 80.5% true prediction ratios, respectively (Extended Data Fig. 9a–c and Supplementary Table 8). The relatively low true prediction ratio of the SAS samples from the Dhakan et al. dataset was possibly due to both the small number of bases and the complex genetic features of the SAS population^24^. From these results we concluded that our likelihood score-based method could predict ancestries based on the human reads in the gut metagenome data.

### Genotype imputation from ultra-deep gut metagenome data

Our likelihood score-based approaches efficiently reconstructed personal information from the human reads in the gut metagenome data. However, whether we could perform definitive variant calling from faecal samples needed to be addressed because genome-wide genetic variant information is highly confidential information that can identify a person and reveal disease risks of individuals. Two-step imputation can efficiently reconstruct genome-wide variant information from low-depth sequencing data and is expected to be useful for low-cost genotyping and ancient genome analyses^25,26^. Therefore, we hypothesized that we could reconstruct whole germline genotypes of common variants from deep-sequenced gut metagenome data with ≥0.5× human genome coverage.

**Fig. 5:**
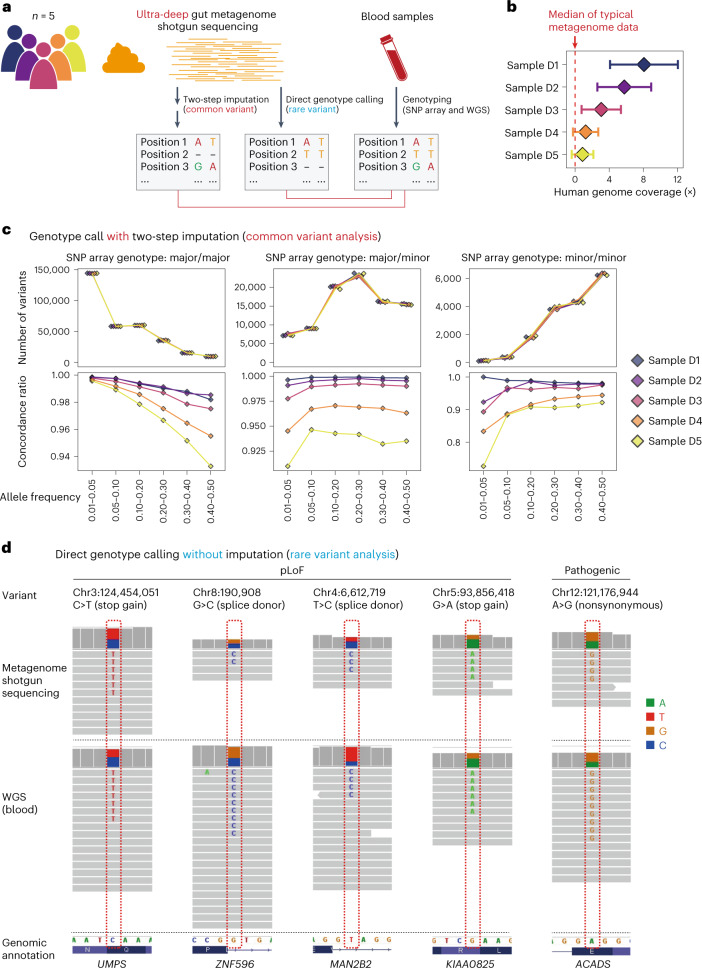
Reconstruction of genotypes from ultra-deep gut metagenome data. **a**, Schematic of the genotype reconstruction from ultra-deep gut metagenome data. The human reads were extracted from ultra-deep gut metagenome data. A genotype call was then performed with or without imputation. The resulting genotypes were subjected to a concordance check with the genotypes obtained from blood samples. **b**, Human genome coverages of the extracted human reads in the ultra-deep gut metagenome data. The error bars indicate the per-sample s.d. across the genomic positions, which were calculated using the CollectWgsMetrics function of the Genome Analysis Toolkit (GATK). The median human genome coverage of the gut metagenome data of 343 Japanese individuals (not ultra-deep) is indicated. **c**, Concordance check between the genotypes obtained from the ultra-deep gut metagenome data with imputation and genotypes obtained from the blood samples based on the SNP array. The number of variants (top) and concordance ratio (bottom) per MAF bin, stratified according to the genotypes in the SNP array, are indicated. **d**, Read mapping to the human reference genome at the pLoF and pathogenic variant sites that were correctly called from the ultra-deep gut metagenome data. Only the sample-specific sites among each WGS dataset are indicated for the pLoF variant sites. The human reads extracted from the ultra-deep gut metagenome (top) and the WGS (middle) data are indicated with genomic annotations (bottom).

To address this, we performed ultra-deep metagenome shotgun sequencing of five faecal samples (Fig. 5a). We sequenced 1.7–2.7 × 10^9^ paired-end reads with data sizes of 252–402 gigabytes, resulting in human genome coverages of 0.88–8.1× (Fig. 5b and Supplementary Table 9). Using the human reads in the ultra-deep gut metagenome data, we performed a two-step genotype imputation. In the first step, the genotype likelihoods were calculated and updated based on the reference genome data. In the second step, genotypes at all of the remaining untyped sites were imputed based on the reference genome data. We checked for concordance with the genotypes obtained from blood samples based on the SNP array (Fig. 5c and Supplementary Table 10). The concordance ratio reached 99% for the samples with a large coverage of the human genome. We also confirmed concordance with the WGS-based genotypes for the disease-associated SNPs (>96.9%; inflammatory bowel diseases^27^ and type 2 diabetes^28^; Supplementary Fig. 9). Thus, we revealed that two-step imputation could efficiently reconstruct the genotypes of the genome-wide common variants from ultra-deep gut metagenome data.

### Rare-variant calling from ultra-deep gut metagenome data

Although the imputation procedure enabled us to reconstruct the genotypes of the common variants, it was technically challenging to obtain the genotypes of the rare variants using the imputation procedures. Therefore, we directly called variants from the human reads in the ultra-deep gut metagenome data using HaplotypeCaller^29^ (Fig. 5a). Given that the genotype call at the low-coverage sites was erroneous (Supplementary Fig. 10a), we set the thresholds for the allelic depth as a part of variant quality control procedures (Methods). Although the number of the originally called variants was strongly dependent on the human read coverages, the relationship between the per-site sequencing depth and concordance ratio was almost consistent across samples (Supplementary Fig. 10b,c).

**Extended Data Fig. 10:**
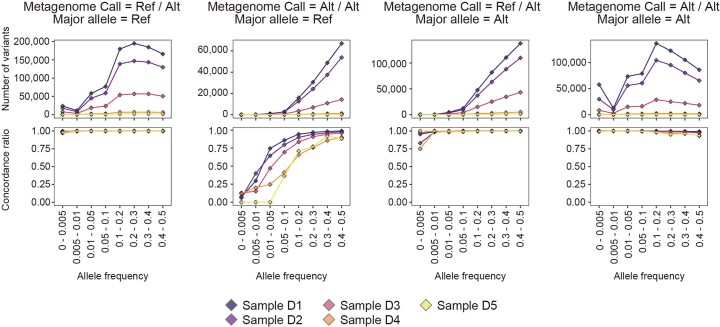
Concordance check of the variants called from the ultra-deep gut metagenome shotgun sequencing data without imputation. Results of the concordance check between the QCed genotypes obtained from the ultra-deep gut metagenome data without imputation and the genotypes obtained from the WGS data. The number of the variants (top) and the concordance ratio (bottom) per MAF bin are indicated stratified according to the genotype calls in the ultra-deep gut metagenome. Alt, alternative allele; DP, depth; QC, quality control; Ref, reference allele.

After variant quality control we obtained 17,268–2,138,159 variants called from the ultra-deep gut metagenome data that had the corresponding genotypes obtained from the WGS data. The number of quality-controlled variants was strongly dependent on the human genome coverage (Extended Data Fig. 10 and Supplementary Table 11). We checked the concordance with the genotypes called from the WGS data and found that the overall concordance ratio was consistently high (99.6–98.9%). Even when stratified according to the MAFs, the concordance ratio for the heterozygous sites, including rare-variant sites, was consistently high (97.0–100.0% when the major allele was the reference allele and 75.0–100.0% when the major allele was the alternative allele; Extended Data Fig. 10 and Supplementary Table 11). A relatively low concordance ratio was observed for the homozygous rare allele (0–12.9% when the major allele was the reference allele and MAF ≤ 0.005), which could be due to the low prior probability of the homozygous of the rare allele. We then evaluated whether the rare putative loss of function (pLoF) variants^17^ (MAF < 0.01) called from the WGS data could also be called from the ultra-deep gut metagenome data. Although we could not call any pLoF variants for the samples with relatively low human read coverage, we successfully called 71.4% (5/7), 50.0% (4/8) and 50.0% (5/10) of the pLoF variants for the samples D1, D2 and D3, respectively (Supplementary Table 12). Note that some of the called pLoF variants were specific to samples in each WGS dataset as well as rare variants or variants not included in the Genome Aggregation Database, which means that this was highly confidential information (Fig. 5d and Supplementary Table 13). We also evaluated the rare pathogenic variants annotated by ClinVar^30^ and found that a rare pathogenic variant in the *ACADS* gene (p.Glu340Gly for deficiency of butyryl-CoA dehydrogenase) was successfully identified from the ultra-deep gut metagenome data for the D2 sample (Fig. 5d and Supplementary Table 13). Thus, we successfully identified a subset of rare variants including pLoF and pathogenic variants from the ultra-deep gut metagenome data.

## Discussion

Although reconstruction of personal information in gut metagenome data is important in terms of the protection of the privacy of the donor and effective utilization of data, a lack of suitable methodology had hampered us from addressing this point. Here we developed a series of methods to quantitatively evaluate how much personal information we could reconstruct from human reads in gut metagenome data. We successfully predicted the genetic sex (97.3%) and ancestry (98.3%) of individuals, and re-identified individuals (93.3%). These results demonstrated the effectiveness of the likelihood score-based method. To evaluate whether we could reconstruct genotypes from gut metagenome data, we performed ultra-deep gut metagenome shotgun sequencing, followed by two-step imputation and direct variant calling. We successfully reconstructed genome-wide common variants using two-step imputation and a subset of clinically impactful rare variants by direct variant calling.

Sex prediction based on DNA in faecal samples had been conducted mainly for wild animals through PCR amplification of marker genes^31,32^. However, the performance of sex prediction based on human gut metagenome data had not been evaluated. Sex prediction based on gut metagenome data could be useful to remove mislabelled samples in human microbiome studies, as done in the field of human genetics^33^.

Our likelihood score-based method quantitatively demonstrated the identifying power of gut metagenome data. The likelihood score-based method had the power to scale to biobank-scale data and its type 1 error could be easily controlled by introducing multiple-test correction to the significance thresholds. Therefore, identification of the sample overlap between the gut metagenome datasets and biobank-scale genotype data is in theory feasible, although it should not be done. Given the importance of protecting the privacy of donors^34,35^, we should correctly understand the identifying power of gut metagenome data and consider human read removal before deposition. We should also understand that identifiable information may remain in gut metagenome data even after human read filtering in some exceptional cases. Although false positives associated with family data could be a potential limitation of our method, it also meant that familial relationships could be inferred from gut metagenome data, which might raise additional ethical concerns. In addition to ethical concerns, our re-identification method could be useful for the quality control of multi-omics datasets with both gut metagenome and human germline genotype data, and forensics based on stool samples^13^.

The likelihood score-based method was also useful for ancestry prediction. Given that differences in morbidity among different genetic ancestries have been reported for many diseases (for example, type 2 diabetes and Crohn’s disease)^36^, genetic ancestral information could reveal highly confidential information such as the disease risk of an individual. In our method ancestries were defined based on the 1KG dataset. Although it is a widely used genome dataset for studying genetic diversity across populations, heterogeneity within some of the populations would still not be fully covered. For example, SAS samples were often misclassified as AMR and EUR, especially when the number of reads was small. This could be because the genetic diversity of the SAS population is complex due to the presence of multiple socio-linguistic groups, founder effect and endogamy along caste and tribal lines^24^, and is not fully reflected in the 1KG dataset (Supplementary Note 4). Ongoing efforts to expand the diversity of the genomic datasets are promising to resolve this limitation^37,38^.

The common variant information reconstructed from the ultra-deep gut metagenome data by the two-step imputation was accurate and supposed to be applicable for other genetic analyses such as polygenic risk score calculation. A subset of the rare variants recovered by direct genotype calling was associated with diseases (that is, pLoF variant in *UMPS* for orotic aciduria and missense variant in *ACADS* for deficiency of butyryl-CoA dehydrogenase^39^), suggesting that the rare-variant information reconstructed from the gut metagenome data could reveal the disease risk of an individual. Given the volume of faecal samples used for ultra-deep sequencing, approximately 40 mg of faeces could be sufficient to call genome-wide common variants and some highly confidential rare variants. Although ultra-deep metagenome shotgun sequencing is not widespread at present, it can be useful for obtaining information on rare microbes and may be performed more frequently in the future if sequencing costs continue to decrease^14^. In addition, these findings could also apply to the sets of multiple gut metagenome data from the same individuals^15,40,41^ and human read-rich gut metagenome data such as those from patients with inflammatory bowel disease and colorectal cancer^5^. Therefore, we should be aware that stool samples can be a source of the germline genome.

Stool samples have been rarely recognized as a useful source of germline genomes in humans but are often used for wild and domestic animals (Supplementary Note 5)^42–45^. Therefore, further development of methods to efficiently utilize the human genome in gut metagenome data could also be also beneficial for animal research.

In summary, we reconstructed personal information from the human reads in gut metagenome data using optimized methods. Our results will be a useful resource to consider the best practice for the deposition of gut metagenome data and contribute to the healthy and sustainable progression of human microbiome research.

## Methods

### Subject participation

The study protocol was approved by the ethics committees of Osaka University and related medical institutions as well as the Translational Health Science and Technology Institute (Faridabad). Japanese individuals (*n* = 343) for whom gut metagenome shotgun sequencing were performed in previous studies were included in this study^46–48^. Among these individuals, 14 and 4 were used in the WGS analysis using HiseqX (Illumina) and NovaSeq 6000 (Illumina) systems, respectively. Among the individuals with WGS data, five were recruited for the ultra-deep metagenome shotgun sequencing analysis. Another 113 Japanese individuals for whom gut metagenome shotgun sequencing had been performed in previous studies^46,49^ were included in the validation dataset for the genetic sex prediction analysis. We newly recruited 37 individuals of various nationalities from Osaka University and the RIKEN Center for Integrative Medical Sciences, and included them in the multi-ancestry dataset for the validation of ancestry prediction analysis. Samples from individuals in Delhi (India) were also included in the multi-ancestry dataset^50^. All study participants provided written informed consent before participation. The main, validation and multi-ancestry datasets included 343, 113 and 73 study participants, respectively, comprised of 196, 65 and 25 females, and 147, 48 and 48 males, respectively. The age ranges for these three datasets were 20–88 yr (mean, 42.2 yr; s.d., 17.3 yr), 20–81 yr (mean, 39.1 yr; s.d., 17.3 yr) and 20–61 yr (mean, 30.5 yr; s.d., 7.9 ), respectively. Summaries of each dataset are provided in Supplementary Table 1. No statistical methods were used to pre-determine sample sizes but our sample sizes are similar to those reported in previous publications^46,47^. The study participants did not receive any compensation.

### Sample collection and DNA extraction

Phenol–chloroform DNA extraction was newly performed in this study for the ultra-deep and multi-ancestry gut metagenome datasets. For the other datasets, phenol–chloroform DNA extraction and subsequent metagenome shotgun sequencing had been performed in previous studies^46–49^. For dataset 2 as well as the ultra-deep gut metagenome dataset and validation dataset for the genetic sex prediction, faecal samples were collected in tubes containing RNAlater (Ambion). After the weights of the samples were measured, RNAlater was added to make tenfold dilutions of the homogenates. The faecal samples were stored at −80 °C within 24 h of collection. After washing with 1 ml PBS(−), 200-μl volumes of the homogenates were used for DNA extraction.

For datasets 1 and 3 as well as the multi-ancestry gut metagenome dataset, faecal samples were stored at −80 °C within 6 h of collection or frozen in an insulated container for storage at −20 °C immediately after collection and subsequently stored at −80 °C within 24 h. For these samples stored without RNAlater, RNAlater was added to make tenfold dilutions of homogenates before the DNA extraction. After washing with 1 ml PBS(−), 200-μl volumes of the homogenates were used for DNA extraction.

DNA was extracted according to a previously described method^51^. Briefly, 300 μl SDS–Tris solution, 0.3 g glass beads (diameter, 0.1 mm; BioSpec) and 500 μl EDTA–Tris-saturated phenol were added to the suspension, and the mixture was vortexed vigorously using a FastPrep-24 homogenizer (MP Biomedicals) at a power level of 5.0 for 30 s. After centrifugation at 20,000*g* for 5 min at 4 °C, 400 μl supernatant was collected. Subsequently, phenol–chloroform extraction was performed and 250 μl of the supernatant was subjected to isopropanol precipitation. Finally, the DNA was suspended in 100 μl EDTA–Tris buffer and stored at −20 °C.

For 88 samples in dataset 1 and 47 samples in the multiple-ancestry dataset, we performed DNA extraction from frozen unhomogenized stool samples using the DNeasy PowerSoil Kit (QIAGEN) as per the manufacturer’s instructions.

### Metagenome shotgun sequencing

The amounts of stool-derived DNA used for the library preparation are provided in Supplementary Table 14. Double-stranded DNA was quantified using a Qubit Fluorometer (Thermo Fisher Scientific). After sonication using an ME220 instrument (Covaris; target sonication length, 200 bp), a shotgun sequencing library was constructed using a KAPA hyper prep kit (KAPA Biosystems) and LifeECO version 2.0 (BIOER Technology) following the manufacturers’ instructions. The library quality was evaluated using a LabChip GX Touch system (PerkinElmer). A Qubit fluorometer and KAPA library quantification kits (KAPA Biosystems) were used to quantify the library and 150-bp paired-end reads were generated on a HiSeq 3000 or NovaSeq 6000 system. Detailed information on the number of lanes, total number of paired-end reads, sequencers, library preparation reagents and library preparation kits for the newly and previously obtained datasets is provided in Supplementary Table 14. The samples in each dataset were barcoded, pooled in equimolar ratios (targeted final amount of the pooled library, 4 nM) and sequenced simultaneously in a single run without library replication. A total of 1.8 nM (in 30 μl) of the pooled library was loaded to the sequencing machine for each sequencing run. All sequencing was performed at the Department of Infection Metagenomics, Next-Generation Sequencing Core Facility, Research Institute for Microbial Diseases, Osaka University. Details of the reagents and kits used for metagenome shotgun sequencing are provided in Supplementary Data. The sequence reads were converted to FASTQ format using bcl2fastq (version 2.19).

### Genotyping of samples based on the SNP array

We performed genotyping using an Infinium Asian screening array (Illumina). This genotyping array was built using an EAS reference panel including whole-genome sequences, which enabled effective genotyping in EAS populations.

We applied stringent quality control filters to the genotyping dataset using PLINK (version 1.90b4.4)^52,53^. We excluded individuals with a genotyping call rate of <0.98. All of the individuals were determined to be of EAS ancestry, based on the principal component analysis (PCA) with the samples of the 1KG dataset using EIGENSTRAT^54^. We further excluded SNPs with a (1) call rate < 0.99, (2) minor allele count < 5 and (iii) Hardy–Weinberg equilibrium *P* value of <1.0 × 10^−5^.

We performed genome-wide genotype imputation to estimate untyped variants computationally. We used the combined reference panel of the 1KG Project Phase 3 version 5 genotype (*n* = 2,504) and Japanese WGS data (*n* = 1,037)^55,56^ as a haplotype reference for genotype imputation. First, we excluded SNPs with >7.5% allele frequency difference to the representative reference datasets of Japanese ancestry, namely the Japanese data in the aforementioned combined reference panel^55,56^ (*n* = 104 from the 1KG Project Phase 3 version 5 genotype and *n* = 1,037 from the Japanese WGS data) and the allele frequency panel of the Tohoku Medical Megabank Project^57^ (ToMMo 8.3KJPN Allele Frequency Panel, *n* = 8,380). Second, we conducted haplotype estimation to improve imputation performance using SHAPEIT (version 4.2.1)^58^ with haplotype reference. After the prephasing, we used Minimac4 (version 1.0.1)^59^ for genotype imputation. The variants imputed with Rsq (estimated value of the squared correlation between the true and imputed genotypes) > 0.7 were used for the downstream analysis.

### Genotyping of samples based on WGS

DNA samples isolated from whole blood were sequenced at Macrogen Japan Corporation. The DNA was quantified using Picogreen and degradation of DNA was assessed by gel electrophoresis. All libraries were constructed using a TruSeq DNA PCR-free library preparation kit according to the manufacturer’s protocol. The libraries were sequenced on a HiSeqX or NovaSeq 6000 system, producing paired-end reads 2 × 150 bp in length, with a mean coverage of 15.5× and 16.4×, respectively. The reads produced by the HiSeqX and NovaSeq 6000 systems were processed as previously described^60^. Briefly, sequenced reads were aligned against the reference human genome with the decoy sequence (GRCh37, human_g1k_v37_decoy) using BWA-MEM (version 0.7.13). Duplicated reads were removed using Picard MarkDuplicates (version 2.10.10). After base-quality score recalibration implemented in GATK (versions 3.8-0), we generated individual variant call results using HaplotypeCaller and performed multi-sample joint-calling of the variants using GenotypeGVCFs. We set genotypes satisfying any of the following criteria as missing: (1) depth of coverage (DP) < 5, (2) genotype quality (GQ) < 20 or (3) DP > 60 and GQ < 95. Variants with low genotyping call rates (<0.90) were then removed. We performed variant quality score recalibration for SNPs and short indels according to the GATK Best Practice recommendations and adopted the variants that passed the quality control criteria. For the annotation of the pLoF variant, we used loftee^17^ in VEP (version 102)^61^ with GENCODE (release 19) annotation and only high-confidence LoF variants were extracted. For the annotation of the ClinVar pathogenic variants, we used ANNOVAR (8 Jun 2020)^62^ with the clinvar_20210123 annotation on GRCh37.

### Extraction of human reads from metagenome data

We followed a series of steps to extract human reads from metagenome data. The main steps in the human read extraction were as follows: (1) trimming of low-quality bases, (2) identification of candidate human reads, (3) removal of duplicated reads and (4) removal of the potential bacterial reads. We trimmed the raw reads to clip Illumina adaptors and cut off low-quality bases using Trimmomatic (version 0.39; parameters: ILLUMINACLIP:TruSeq3-PE-2.fa:2:30:10:8:true TRAILING:20 MINLEN:60)^63^. We discarded reads that were smaller than 60 bp in length after trimming. Next, we mapped the trimmed reads to the human reference genome (GRCh37, human_g1k_v37_decoy) using Bowtie 2 (version 2.3.5.1)^64^ with the ‘—no-discordant’ option and retained only the properly mapped reads. We then performed duplicate removal using Picard MarkDuplicates (version 2.22.8) with the ‘VALIDATION_STRINGENCY = LENIENT’ option. Finally, we mapped the duplicate removed reads to the bacterial reference genome set constructed by Kishikawa and colleagues^65^. This reference was composed of the 7,881 genomes including those derived from Nishijima et al.^66^ and those identified in the cultivated human gut bacteria projects^67–69^. We only kept reads for which both paired ends failed to align. The resulting reads were defined as human reads and used in the subsequent analyses. The read counts obtained using bedtools (version 2.29.2) was normalized to the number of reads to adjust for differences in sequencing depths between samples. The normalized read count was further normalized to the chromosome length to calculate the normalized depth.

### Evaluation of the quality of the human reads in gut metagenome data

We utilized FastQC^70^ to assess the adaptor content and GC ratio of the reads. Based on the outputs from the ‘bcftools mpileup’, the transition to transversion SNP ratio of the reads was estimated for the non-reference bases. We utilized ‘bcftools mpileup’ to evaluate the ratio of the non-major allele on the mitochondrial genome, which could be associated with the contamination of non-host human DNA. We also used mixemt (version 0.2) to evaluate the ratio of the non-major haplogroup, which could also be associated with non-host human DNA contamination^71^. To evaluate the performance of the methods for estimating non-host human DNA contamination, we simulated mtDNA-derived human reads using WGS data of five individuals. We made use of WGS data of another five individuals as sources of contaminated mtDNA-derived reads. Next, we mixed the two mtDNA reads derived from the WGS data—one from the original sample set and one from the contamination dataset—in specific ratios (original:contamination of 100:0, 90:10, 75:25 and 50:50) and downsampled to 1, 2, 5, 10, 15 and 20× human mitochondrial genome coverages in five seeds using SAMtools^72^. We then applied the above two methods to the simulated data and estimated the levels of non-host human DNA contamination. After the simulation experiment, we estimated the levels of non-host human DNA contamination in the gut metagenome data of 343 Japanese individuals.

### Genetic sex prediction based on the human reads in the gut metagenome data

Using scikit-learn (version 0.22.1), we trained a logistic regression model to predict genetic sex (male = 1 and female = 0) based on the Y-to-X chromosome read-depth ratio. Note that we used only the non-pseudo-autosomal regions for this analysis. We then validated this model with the validation dataset (*n* = 113). We also performed the same analysis after the human read extraction with the different human reference genomes, namely GRCh38 (GCA_000001405.15_GRCh38_no_alt_analysis_set) and T2T-CHM13v2.0 (chm13v2.0_maskedY_rCRS).

### Re-identification from a set of genotype data based on the human reads in the gut metagenome data

We calculated the likelihood that each sample in the genotype dataset produced the observed human reads in the faecal samples from two input data—that is, the human reads in the gut metagenome data that were mapped to the human reference genome and the genotype dataset (imputed SNP array data were used in this study) of the multiple samples.

We extracted the SNP sites that were covered by at least a read and included in the reference panel by ‘bcftools mpileup’ with the ‘-T’ option. In this study we used a combined reference panel of the 1KG Project Phase 3 version 5 genotype (*n* = 104) and Japanese WGS data (*n* = 1,037) as the reference panel. To get independent SNP sites, we applied clumping to the list of the SNPs that were covered by at least a read. We used the ‘–indep-pairwise 100 30 0.1’ option in PLINK for clumping at Rsq = 0.1. We then calculated the likelihood according to the model proposed in Li and colleagues^72^. Suppose an SNP site *i* was covered by *n*_i_ reads in the gut metagenome data, *k*_i_ reads were from the reference allele and *n*_i_ − *k*_i_ reads were from the alternative allele. In this study bcftools (version 1.15.1) was used to calculate the read coverage with the ‘-q 40 -Q 20’ option. The error probability of the read bases was *ε* and error independency was assumed. In this study *ε* was set at 1 × 10^−6^ following the previously reported assumption^72^. At the SNP site *i*, the number of alternative alleles of an individual *j* (*g*_i,j_) could be zero (Ref/Ref), one (Ref/Alt) or two (Alt/Alt), where Ref is the reference allele and Alt is the alternate allele. The likelihood (*L*) that the sample with *g*_i_ alternative alleles at SNP site *i* produced the observed human reads in the gut metagenome data was expressed as

$$L_{\mathrm{i,j}}\left( {g_{\mathrm{i,j}},n_\mathrm{i},k_\mathrm{i}} \right) = \frac{1}{{2^{n_\mathrm{i}}}}\left[ {\left( {2 - g_{\mathrm{i,j}}} \right)\varepsilon + g_{\mathrm{i,j}}\left( {1 - \varepsilon } \right)} \right]^{n_\mathrm{i} - k_\mathrm{i}}\left[ {\left( {g_\mathrm{{i,j}}\varepsilon + \left( {2 - g_\mathrm{{i,j}}} \right)\left( {1 - \varepsilon } \right)} \right.} \right]^{k_\mathrm{i}}$$

When the clumping procedure retained *N* independent SNP sites, a log-transformed likelihood (likelihood score, *LS*) that a genotype data produced the observed human reads in the gut metagenome data was expressed as

$$LS_\mathrm{j} = \mathop {\sum }\limits_{i = 1}^N {{{\mathrm{log}}}}(L_{\mathrm{i,j}}(g_\mathrm{{i,j}},n_\mathrm{i},k_\mathrm{i}))$$

Next, we drew the background distribution of the likelihood score from (1) human reads in the gut metagenome data that were mapped to the human reference genome and (2) allele frequency data for the SNP sites used for calculating the likelihood score. Note that the ancestry of the allele frequency data should be matched to the samples. In this study Japanese individuals in the combined reference panel of the 1KG Project Phase 3 version 5 genotype (*n* = 104) and Japanese WGS data (*n* = 1,037) were used to calculate the allele frequency. When an alternative allele frequency at SNP site *i* was *p*_i_ and the number of the alternative allele was *g*_i,pop_ (= 0, 1 or 2), the theoretical genotype frequencies at SNP site *i* were expressed as

$$P\left( {g_{\mathrm{i,pop}},p_\mathrm{i}} \right) = \left\{ {\begin{array}{*{20}{c}} {(1 - p_\mathrm{i})^2,} & {(g_{\mathrm{i,pop}} = 0)} \\ {2p_\mathrm{i}\left( {1 - p_\mathrm{i}} \right),} & {(g_{\mathrm{i,pop}} = 1)} \\ {p_\mathrm{i}^2,} & {(g_{\mathrm{i,pop}} = 2)} \end{array}} \right.$$

The expected log-transformed likelihood that a genotype data randomly drawn from the specified population produced the observed human reads in the metagenome data was then expressed as

$$E\left( {LS_{\mathrm{pop}}} \right) = \mathop {\sum }\limits_{i = 1}^N E\left( {LS_{\mathrm{i,pop}}} \right) = \mathop {\sum }\limits_{i = 1}^N \mathop {\sum }\limits_{g_{\mathrm{i,pop}} = 0}^2 P\left( {g_{\mathrm{i,pop}},p_\mathrm{i}} \right){{{\mathrm{log}}}}(L_\mathrm{i}(g_{\mathrm{i,pop}},n_\mathrm{i},k_\mathrm{i}))$$

Given that the SNP sites were independent, the variance (*V*) of the likelihood score in a specific population was expressed as

$$\begin{array}{l}V\left( {LS_{\mathrm{pop}}} \right) = \mathop {\sum }\limits_{i = 1}^N V\left( {LS_{\mathrm{i,pop}}} \right) =\\ \mathop {\sum }\limits_{i = 1}^N \mathop {\sum }\limits_{g_{\mathrm{i,pop}} = 0}^2 P\left( {g_{\mathrm{i,pop}},p_\mathrm{i}} \right)[{{{\mathrm{log}}}}(L_\mathrm{i}\left( {g_{\mathrm{i,pop}},n_\mathrm{i},k_\mathrm{i}} \right)) - E\left( {LS_{\mathrm{i,pop}}} \right)]^2\end{array}$$

Using *E*(*LS*_*pop*_) and *V*(*LS*_*pop*_), we calculated the standardized likelihood score of the individual *j* as $(LS_\mathrm{j} - E\left( {LS_{\mathrm{pop}}} \right))/\sqrt {V\left( {LS_{\mathrm{pop}}} \right)}$.

From the experiment with the simulated and real data, we found that the standardized likelihood score was almost normally distributed when the read and genotype data originated from different samples (Extended Data Figs. 3a and 5c). Therefore, we transformed standardized likelihood scores to *P* values based on a normal distribution. Empirical *P* values were also calculated for *LS*_j_ from the randomly simulated genotype data of the 99,999 SNP sites used for calculating the likelihood score. Genotypes of each site were independently drawn from the allele frequency data and empirical *P* values were defined as: (1 + number of the genotype data with the likelihood score higher than *LS*_j_) / 100,000.

### Re-identification from a set of genotype data based on simulated human read data

To test the performance of the likelihood score-based re-identification, we simulated human reads by downsampling five WGS datasets to human genome coverages of 1 × 10^−5^, 2 × 10^−5^, 5 × 10^−5^, 1 × 10^−4^, 2 × 10^−4^, 5 × 10^−4^ and 1 × 10^−3^× using SAMtools. We generated 25 datasets (5 samples × 5 seeds) for each coverage. We then calculated the likelihood scores using the downsampled read data and imputed SNP array data of 100 samples, which included the five samples used to make downsampled read data. We also simulated human reads using three WGS datasets that had familial relationships and repeated the same experimental procedures to evaluate how the familial relationship affected the likelihood score. As a reference panel for defining the SNP sites and calculating the background distribution of the likelihood score, we used Japanese individuals in the combined reference panel of the 1KG Project Phase 3 version 5 genotype and Japanese WGS data throughout all of the simulation analyses.

Using the same sample set, we performed another simulation experiment using only SNP sites whose MAF was within specific ranges, namely (0, 0.05], (0.05, 0.1], (0.1, 0.2], (0.2, 0.3], (0.3, 0.4] and (0.4, 0.5]. For the unrelated five samples used in the simulation experiment, we simulated human reads by downsampling five WGS datasets to a human genome coverage of 1 × 10^−2^× in five seeds using SAMtools. We then calculated the likelihood score and its background distribution based on the randomly selected 100, 150, 200, 300, 400, 500, 750, 1,000, 1,500 and 2,000 bases on the human reads that covered the SNPs with the specified range of MAF.

We also evaluated how non-host human DNA contamination would impact the re-identification performance using the same sample set. We made use of another five WGS datasets as sources of the simulated contamination. Next, we mixed two sets of WGS data—one from the original sample set and the other from the contamination dataset—in specific ratios (original:contamination of 100:0, 90:10, 75:25, 50:50, 25:75 and 10:90) and downsampled to human genome coverages of 1 × 10^−5^, 2 × 10^−5^, 5 × 10^−5^, 1 × 10^−4^, 2 × 10^−4^, 5 × 10^−4^ and 1 × 10^−3^× with five seeds using SAMtools. Using the simulated contaminated data and the imputed SNP array data of 99 samples, which included the original sample set but excluded the contaminated sample, we conducted the re-identification analysis.

For the simulated human read data, we also performed the simulation analysis with a wide range of genotype data sizes rather than the imputed SNP array data of 100 samples. We randomly extracted 5,000 unrelated individuals (PIHAT < 0.185) from the BioBank Japan (BBJ) second cohort data (https://humandbs.biosciencedbc.jp/hum0311-v2), which were also genotyped using the Infinium Asian screening array. Quality control and genotype imputation of the BBJ second cohort data were simultaneously performed with the genotype data of the 343 in-house samples. Next, we combined the genotype data of 9, 19, 49, 99, 199, 499, 999, 1,999 and 4,999 individuals from the BBJ second cohort data with the genotype data for which the simulated human read data was generated to create a genotyping dataset for each simulated human read data. Finally, we performed the re-identification analysis with the combined genotype dataset and calculated the sensitivity and specificity.

### Re-identification from a set of genotype data based on the human reads in the gut metagenome data of 343 individuals

In the experiment with real data, we used 343 samples that had both the gut metagenome data and imputed SNP array data. As a reference panel for defining the SNP sites and calculating the background distribution of the likelihood score, we used Japanese individuals in the combined reference panel of the 1KG Project Phase 3 version 5 genotype and Japanese WGS data throughout all of the real data analyses. We identified gut metagenome data–genotype data (imputed SNP array data was used in this study) pairs derived from the same individuals based on the following two different methods and evaluated the sensitivity and specificity: (1) genotype data with the highest likelihood score and (2) genotype data with *P* < 4.25 × 10^−7^ (0.05 / (343 × 343)).

We performed the analysis with a wide range of genotype data sizes as performed for the simulated dataset. We randomly extracted the 5,000 unrelated individuals (PIHAT < 0.185) from the BBJ second cohort data, which was also genotyped using the Infinium Asian screening array. Next, we combined the genotype data of 9, 19, 49, 99, 199, 499, 999, 1,999 and 4,999 individuals from the BBJ second cohort data with the genotype data from the individual with the targeted metagenome data to create a genotyping dataset for each metagenome data. Finally, we performed the re-identification analysis with the combined genotype dataset and calculated the sensitivity and specificity.

We performed a re-identification analysis with the human reads in the 343 gut metagenome data after human read filtering. We performed human read filtering using several different software packages—that is, BMTagger, Kraken 2 (ref. ^73^), HISAT2 (ref. ^74^), BWA-MEM^75^, Bowtie 2 (ref. ^64^) and KneadData^76^. All software packages were run using default settings with the reference database constructed from the same human reference genome (GRCh37, human_g1k_v37_decoy). Given that all software packages, excepting KneadData, required read pre-processing, such as adaptor trimming, we used Trimmomatic and Fastp^77^ before human read masking. Trimmomatic was used in the same setting as in the human read-extraction pipeline. Fastp was used with −3 −5 -w --adaptor TruSeq3-PE-2.fa. We first extracted human reads, which were detected by our pipeline, from the original fastq files. For the extracted unprocessed human reads, we evaluated whether they were removed by each human read filtering method. Given that all software packages, excepting KneadData, output only the mapping or classification results, we only kept reads for which both paired ends failed to align to the human reference genome for them. Using the human reads that were retained even after human read filtering, we performed a re-identification analysis in the same setting as described earlier.

### Prediction of the ancestry of gut metagenome data

We calculated the likelihood that the observed human reads in the gut metagenome data from two input data—(1) human reads that were mapped to the human reference genome and (2) allele frequency data of the multiple ancestries—were derived from each ancestry. In this study we used a 1KG dataset (http://ftp.1000genomes.ebi.ac.uk/vol1/ftp/release/20130502/) processed with ‘bcftools view -m2 -M2’ to calculate the allele frequency of the AMR, EUR, AFR, EAS and SAS populations. For the definition of the five ancestries, the following 2,504 samples in the 1KG dataset were used. AMR: Colombians in Medellin, Colombia (*n* = 94); people with Mexican ancestry in Los Angeles, CA, USA (*n* = 64); Peruvians in Lima, Peru (*n* = 85); and Puerto Ricans in Puerto Rico (*n* = 104). EUR: Utah residents (CEPH) with Northern and Western European ancestry (*n* = 99), British individuals in England and Scotland (*n* = 91), Finnish individuals in Finland (*n* = 99), Iberian populations in Spain (*n* = 107) and Toscani in Italy (*n* = 107). AFR: Esan individuals in Nigeria (*n* = 99); Gambian individuals in Western Division, Mandinka (*n* = 113); Luhya individuals in Webuye, Kenya (*n* = 99); Mende individuals in Sierra Leone (*n* = 85); Yoruba individuals in Ibadan, Nigeria (*n* = 108); African Caribbean individuals in Barbados (*n* = 96) and people with African ancestry in Southwest USA (*n* = 61). EAS: Chinese Dai individuals in Xishuangbanna, China (*n* = 93); Han Chinese individuals in Beijing, China (*n* = 103); Southern Han Chinese individuals (*n* = 105), Japanese individuals in Tokyo, Japan (*n* = 104); and Kinh individuals in Ho Chi Minh City, Vietnam (*n* = 99). SAS: Bengali individuals in Bangladesh (*n* = 86); Gujarati Indians in Houston, TX, USA (*n* = 103); Indian Telugu individuals in the UK (*n* = 102); Punjabi individuals in Lahore, Pakistan (*n* = 96) and Sri Lankan Tamil individuals in the UK (*n* = 102). We extracted SNP sites covered with the human read in the gut metagenome data from the allele frequency data.

When an alternative allele frequency at an SNP site *i* in a population *A* is *p*_i,A_ and the number of the alternative allele is *g*_i_, the theoretical genotype frequencies at SNP site *i* in a population *A* are expressed as

$$P\left( {g_\mathrm{i},p_{\mathrm{i,A}}} \right) = \left\{ {\begin{array}{*{20}{c}} {(1 - p_{\mathrm{i,A}})^2,} & {(g_\mathrm{i} = 0)} \\ {2p_{\mathrm{i,A}}\left( {1 - p_{\mathrm{i,A}}} \right),} & {(g_\mathrm{i} = 1)} \\ {p_{\mathrm{i,A}}^2,} & {(g_\mathrm{i} = 2)} \end{array}} \right.$$

In the same setting as in the case of the re-identification from a set of genotype data, the expected value of the likelihood score that the human reads were derived from the population *A* was expressed as

$$E(LS_\mathrm{A}) = \mathop {\sum }\limits_{i = 1}^N E\left( {LS_{\mathrm{i,A}}} \right) = \mathop {\sum }\limits_{i = 1}^N \mathop {\sum }\limits_{g_\mathrm{i} = 0}^2 P\left( {g_\mathrm{i},p_\mathrm{{i,A}}} \right){{{\mathrm{log}}}}(L_\mathrm{i}(g_\mathrm{i},n_\mathrm{i},k_\mathrm{i}))$$

In this study expected likelihood scores were calculated for all five superpopulations in the 1KG dataset, namely AMR, EUR, AFR, EAS and SAS. The superpopulation with the highest expected likelihood score was defined as the predicted ancestry.

For the simulation experiment, we downloaded the WGS data of 250 samples (50 individuals × 5 populations) from the Human Genome Diversity Project (Supplementary Table 6). We randomly extracted 300,000 paired-end reads for each sample, processed with the human read-extraction pipeline and downsampled with scales of 1 × 10^−5^, 2 × 10^−5^, 5 × 10^−5^, 1 × 10^−4^, 2 × 10^−4^, 5 × 10^−4^ and 1 × 10^−3^× human genome coverage. The ancestries of the downsampled read data were then predicted based on the above model and the true prediction ratio was calculated. PCA analysis of the SAS samples in the Human Genome Diversity Project dataset was performed with previously published genotype data^78^ (downloaded from ftp://ngs.sanger.ac.uk/production/hgdp/hgdp_wgs.20190516). The genotype data were merged to the 1KG dataset (downloaded from http://ftp.1000genomes.ebi.ac.uk/vol1/ftp/data_collections/1000G_2504_high_coverage/working/20220422_3202_phased_SNV_INDEL_SV/) and SNPs in the hapmap3 dataset were extracted (downloaded from the GATK Resource Bundle, https://gatk.broadinstitute.org/hc/en-us/articles/360035890811-Resource-bundle). Finally, merged genotype data were subjected to pruning (PLINK --indep-pairwise 50 5 0.8) and PCA (PLINK --pca) with PLINK.

For the in-house Japanese (EAS) and multiple-ancestry (EUR, EAS and SAS) datasets, we used 343 and 73 samples, respectively. In addition, we downloaded three publicly available gut metagenome datasets previously published by Karlsson et al. (EUR; accession number: ERP002469)^21^, Zhu et al, (EAS; accession number: ERP111403)^22^ and Dhakan et al. (SAS; accession number: SRP114847)^23^. The downloaded datasets were processed with the human read-extraction pipeline. Next, the ancestries of the gut metagenome data were predicted with the above model and the true prediction ratio was calculated. The in-house EAS and EUR samples were defined based on self-reported ancestries and their ancestries were confirmed by the genotype PCA plot (Extended Data Figs. 5a and 8d). The ancestries of the in-house SAS samples (India), publicly available datasets (EUR, Sweden; EAS, China; and SAS, India) were based on the origin of countries.

### Two-step genotype imputation from ultra-deep metagenome data

We used a two-step imputation pipeline previously used in Homburger et al.^25^ with slight modifications. First, we calculated the genotype likelihoods of SNPs from the human reads in the ultra-deep metagenome data. The mpileup function (‘-q 20 -Q 20’ options), followed by the call function in bcftools were used for the calculation. An update of the genotype likelihoods based on the reference genome data was performed for the SNP sites covered by at least one read using the genotype likelihood option implemented in BEAGLE 4.1 (ref. ^79^). Finally, the second round of imputation was performed using BEAGLE 5.0 (ref. ^80^) to generate genotypes at all of the remaining untyped sites. We used the combined reference panel of the 1KG Project Phase 3 version 5 genotype and Japanese WGS data as in the case of the genotype imputation of SNP array data. The concordance ratio to the SNP array data (not imputed), stratified according to the genotypes in the SNP array and MAF calculated from the Japanese individuals in the reference panel, was calculated. The concordance ratio to the WGS data was also calculated for previously reported disease-associated SNPs (inflammatory bowel diseases^27^ and type 2 diabetes^28^; *P*_GWAS_ < 5 × 10^−8^). Independent disease-associated SNPs were selected using the --clump command of PLINK (--clump-r2 = 0.1 and --clump-kb = 250). Finally, SNPs genotyped using the WGS analysis were extracted for the concordance check.

### Genotype calling from ultra-deep metagenome data without imputation

We performed variant calling without two-step imputation using GATK (version 4.1.7.0). The human reads in the gut metagenome data that were mapped to the human reference genome were subjected to analysis using HaplotypeCaller with default parameters. Hard filtering with the following parameters was applied: QD < 2.0, DP < 2.0, QUAL < 30.0, SOR > 3.0, FS > 60.0, MQ < 40.0, MQRankSum < −12.5 and ReadPosRankSum < −8.0. We applied the following additional filter for the per-allele depths based on the result of the concordance check with the WGS data (Supplementary Fig. 10a): alternative allele depth ≥ 2 and reference allele depth ≥ 2 for the Ref/Alt call, and alternative allele depth ≥ 6 and reference allele depth = 0 for the Alt/Alt call. Note that Ref/Ref sites were not called in this pipeline because joint-calling was not applicable.

For the variants called in both the WGS data and the ultra-deep metagenome data, the concordance ratio was calculated with stratification based on the called genotypes in the metagenome data and MAF in the WGS dataset. For the visualization of the reads mapped to the variant sites, we used IGV (version 2.12.2)^81^.

### Statistics and reproducibility

No statistical method was used to pre-determine sample size. No data were excluded from the analyses. We did not use any study design that required randomization or blinding.

### Reporting summary

Further information on research design is available in the Nature Portfolio Reporting Summary linked to this article.

## Supplementary information

Supplementary Information Supplementary Figs. 1–10 and Notes 1–5.

Reporting Summary

Supplementary Tables 1–14 Supplementary tables (title and description of the tables are described within the file itself).

Supplementary Data Individual data for the number of the human reads and mtDNA-related metrics. Source data for Supplementary Figs. 1 and 2.

Supplementary Data List of the reagents/kits used in this study with company name and catalogue number information.

## Data Availability

The metagenome shotgun sequencing data generated in this study (multi-ancestry dataset, ultra-deep sequencing dataset and dataset generated using the DNeasy PowerSoil kit) are available at the Japanese Genotype-Phenotype Archive (JGA) under the accession number JGAS000600. The SNP array and WGS data for the human blood used in this study are available at the European Genome–Phenome Archive under the accession number EGAS00001007027. The Japanese metagenome shotgun sequencing data derived from the previous studies (datasets 1–3 and validation dataset) are available at the JGA under the accession numbers JGAS000260, JGAS000316, JGAS000531 and JGAS000415, respectively. The metagenome shotgun sequencing data derived from Zhu and colleagues^22^ are available at the European Nucleotide Archive database under the accession number PRJEB29127. The metagenome shotgun sequencing data derived from Dhakan et al.^23^ are available at the Sequence Read Archive (SRA) database under the accession number PRJNA397112. The metagenome shotgun sequencing data derived from Karlsson and colleagues^21^ are available at the SRA database under the accession number PRJEB1786. The genotype data of the BBJ second cohort are available at the JGA under the accession number JGAS000412. Source data are provided with this paper.

## Source data

Source Data Fig. 2 Statistical source data.
Source Data Fig. 3 Statistical source data.
Source Data Fig. 4 Statistical source data.

## Code availability

Custom scripts used for analyses are in the Human_reads_in_metagenome GitHub repository (https://github.com/ytomofuji/Human_reads_in_metagenome).
