# Supplementary material for: Reconstruction of the personal information from human genome reads in gut metagenome sequencing data
Source: Nat Microbiol. 2023 May 15;8(6):1079–94. doi: 10.1038/s41564-023-01381-3 (PMC10234815; doi:10.1038/s41564-023-01381-3)
Supplement: Supplementary file 1 — Supplementary Figs. 1–10 and Notes 1–5. [file 41564_2023_1381_MOESM1_ESM.pdf]

nature microbiology

Article https://doi.org/10.1038/s41564-023-01381-3

Reconstruction of the personal information from human genome reads in gut metagenome sequencing data

In the format provided by the authors and unedited

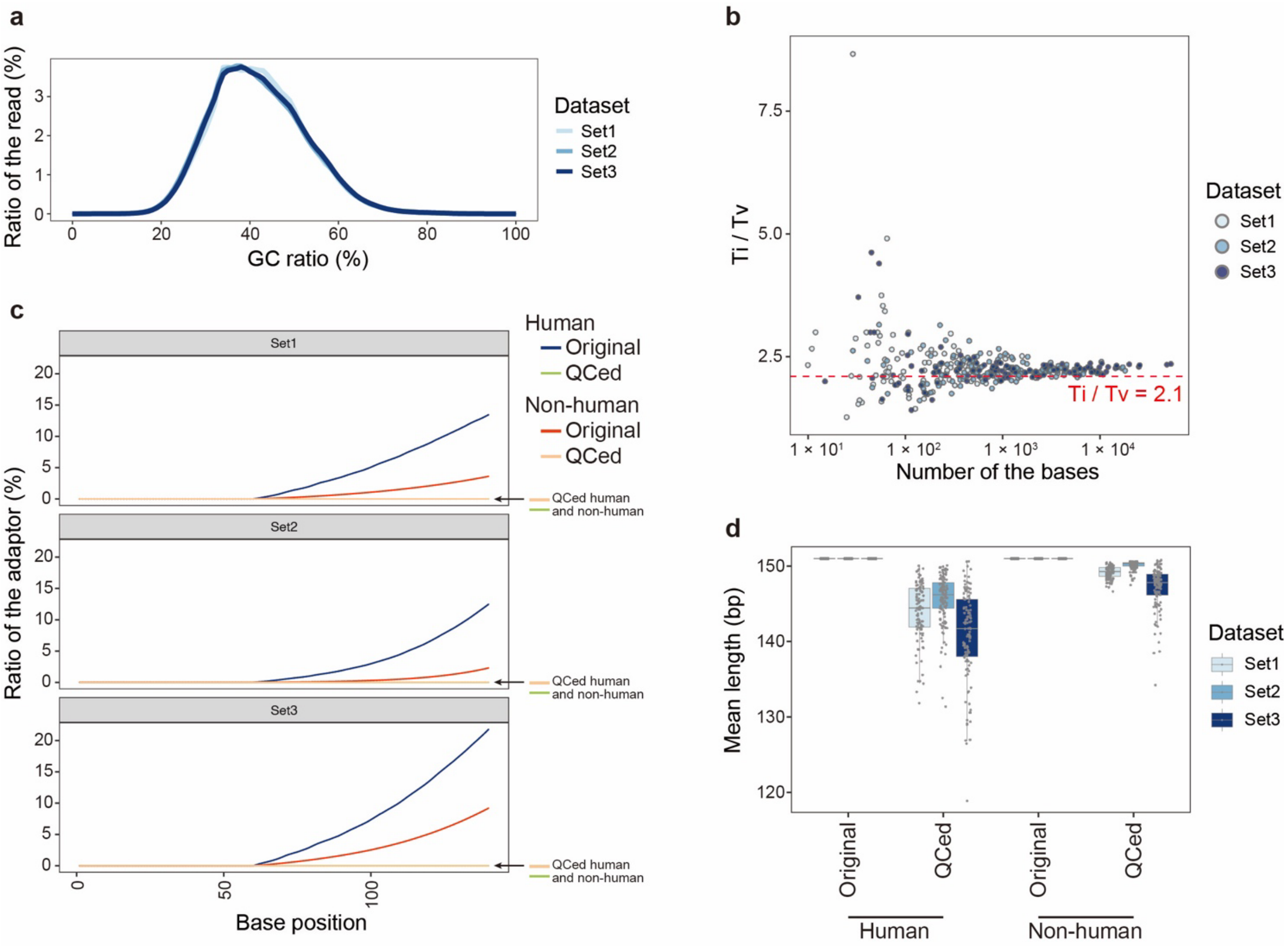

Supplementary Figure 1. Evaluation of the quality of the human reads in the gut metagenome data

a, A plot indicates the distribution of the GC ratio of the human reads in the metagenome data. Mean across the samples included in each dataset is indicated. **b**, A plot indicates the number of the bases in the gut metagenome reads that cover polymorphic sites (x-axis) and the transitions to transversion (Ti / Tv) ratio of the gut metagenome reads (y-axis). A red line indicates the Ti / Tv ratio = 2.1. **c**, Plots indicate the ratio of the adaptor contents per base position. The colors of the line indicate (i) whether the reads are human-derived ones or not and (ii) whether the read QC including adaptor trimming is performed or not. **d**, Boxplots indicate the per-sample mean length of the reads. Reads are stratified based on (i) whether the reads are human-derived ones or not and (ii) whether the read QC including adaptor trimming is performed or not. Boxplots indicate the median values (center lines) and IQRs

(box edges), with the whiskers extending to the most extreme points within the range between (lower quantile − [1.5 × IQR]) and (upper quantile + [1.5 × IQR]). *N* = 102, 129, and 112 biologically independent samples, respectively for datasets 1, 2, and 3. IQR, interquartile ranges; QC, quality control.

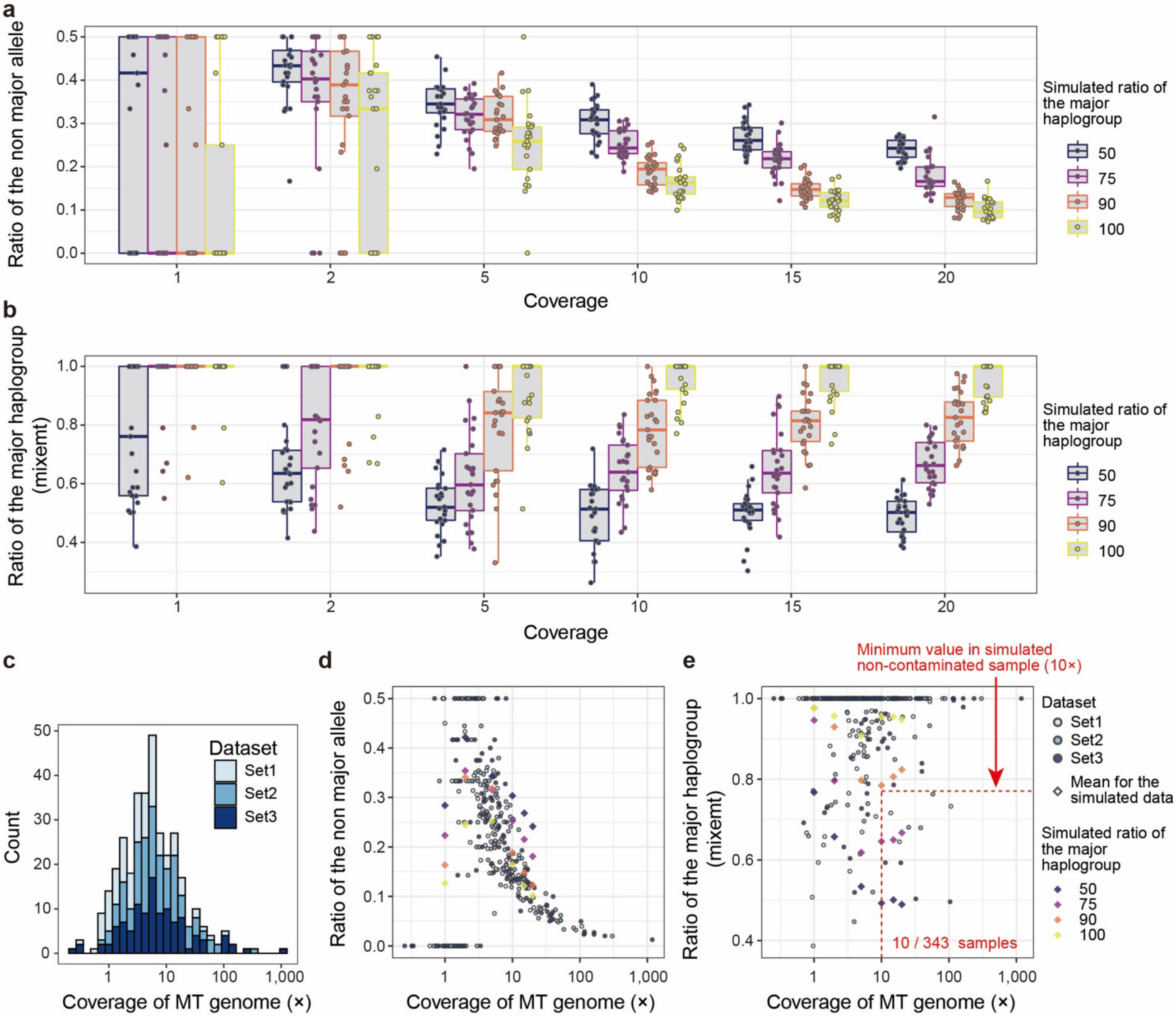

Supplementary Figure 2. Evaluation of the contamination of the non-host human reads based on the mitochondrial reads

a,**b**, Box plots indicate the ratio of the non-major allele (a) and the ratio of the major haplogroup (b) of the mitochondrial DNA. Simulated contaminated data is generated from two human blood whole genome sequencing data (**Methods**). Results for the 600 simulated data (5 sample sets × 5 seeds × 4 simulated ratios of the contamination × 6 coverages) are indicated. Boxplots indicate the median values (center lines) and IQRs (box edges), with the whiskers extending to the most extreme points within the range between (lower quantile − [1.5 × IQR]) and (upper quantile + [1.5 × IQR]). **c**, A histogram indicates the mitochondrial DNA coverages for the 343 gut metagenome data. **d**,**e**, Scatter plots indicate the

mitochondrial DNA coverages (x-axis) and the ratio of the non-major allele (d) and the ratio of the major haplogroup (e) of the mitochondrial DNA (y-axis) for the 343 gut metagenome data. Mean statistics for the simulated contaminated data (4 simulated ratios of the contamination × 6 coverages) are also indicated. IQR, interquartile ranges; MT, mitochondria.

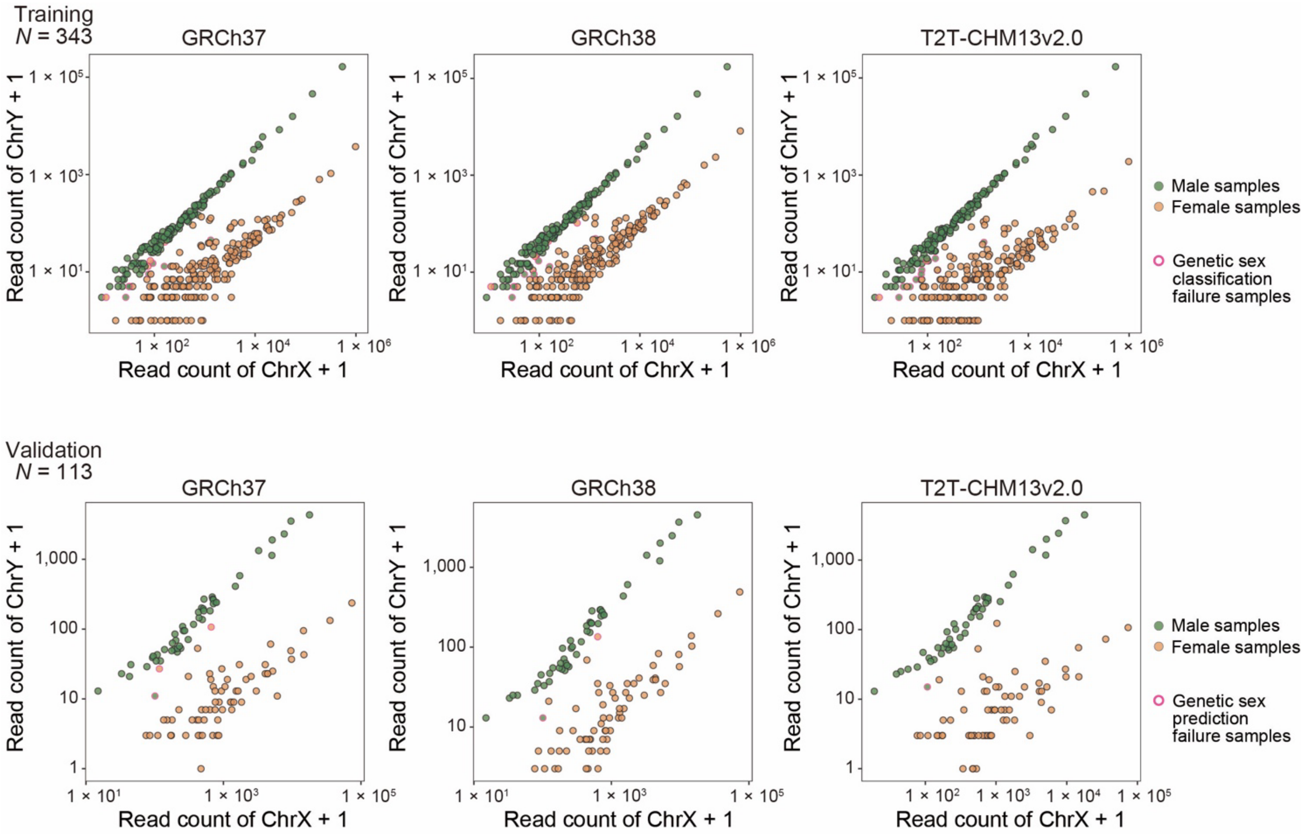

Supplementary Figure 3. The raw number of X chromosomal and Y chromosomal reads in the gut metagenome data

Plots indicate the raw number of X chromosomal (x-axis) and Y chromosomal (y-axis) reads in the gut metagenome data of the training (top) and validation (bottom) datasets. Results for the different human reference genomes (left: GRCh37, middle: GRCh38, right: T2T-CHM13v2.0) are indicated. The colors of the fills represent the genetic sex of the samples. The colors of the outlines represent whether the sex prediction from the human reads extracted from the metagenome data is correct or not.

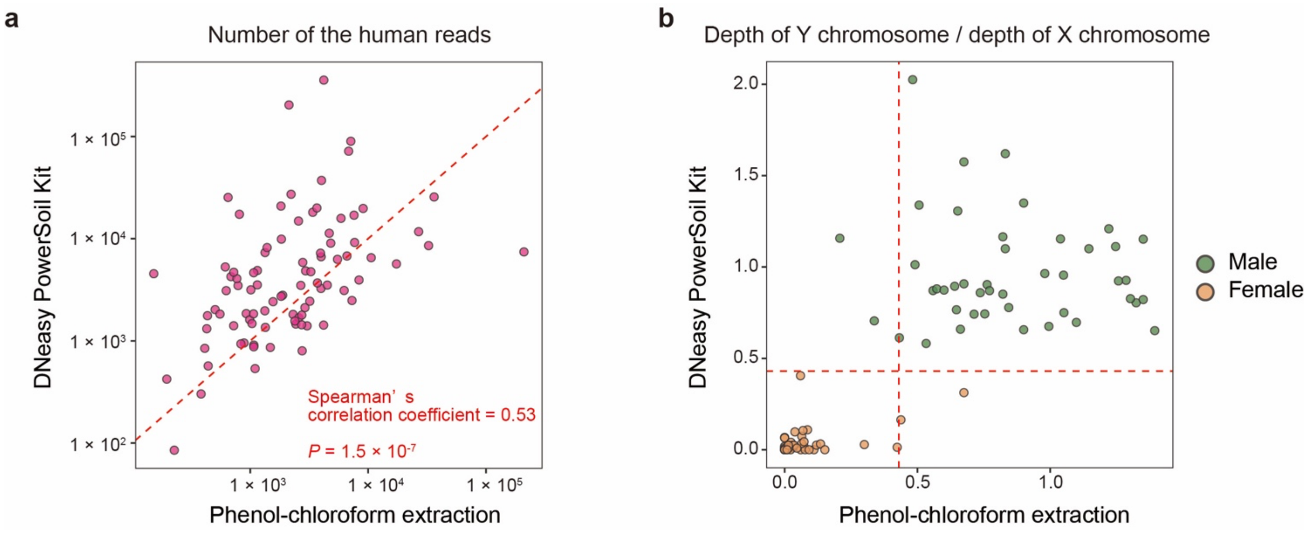

Supplementary Figure 4. Comparisons of the Y chromosomal to X chromosomal read depth ratio between DNA extraction methods

a, A scatter plot represents the number of human reads for the metagenome data obtained from the same 88 samples (a subset of samples in the dataset 1) through the DNA extraction method used in the main analyses (phenol-chloroform extraction, x-axis) and kit-based DNA extraction (DNeasy PowerSoil Kit, y-axis). A red dashed line represents $x = y$. **b**, A scatter plot represents the ratio of the Y chromosomal read depth to the X chromosomal read depth. Results for the metagenome data obtained from the same 88 samples through the DNA extraction method used in the main analyses (phenol-chloroform extraction, x-axis) and kit-based DNA extraction (DNeasy PowerSoil Kit, y-axis) are indicated. A red dashed line represents $x = y$.

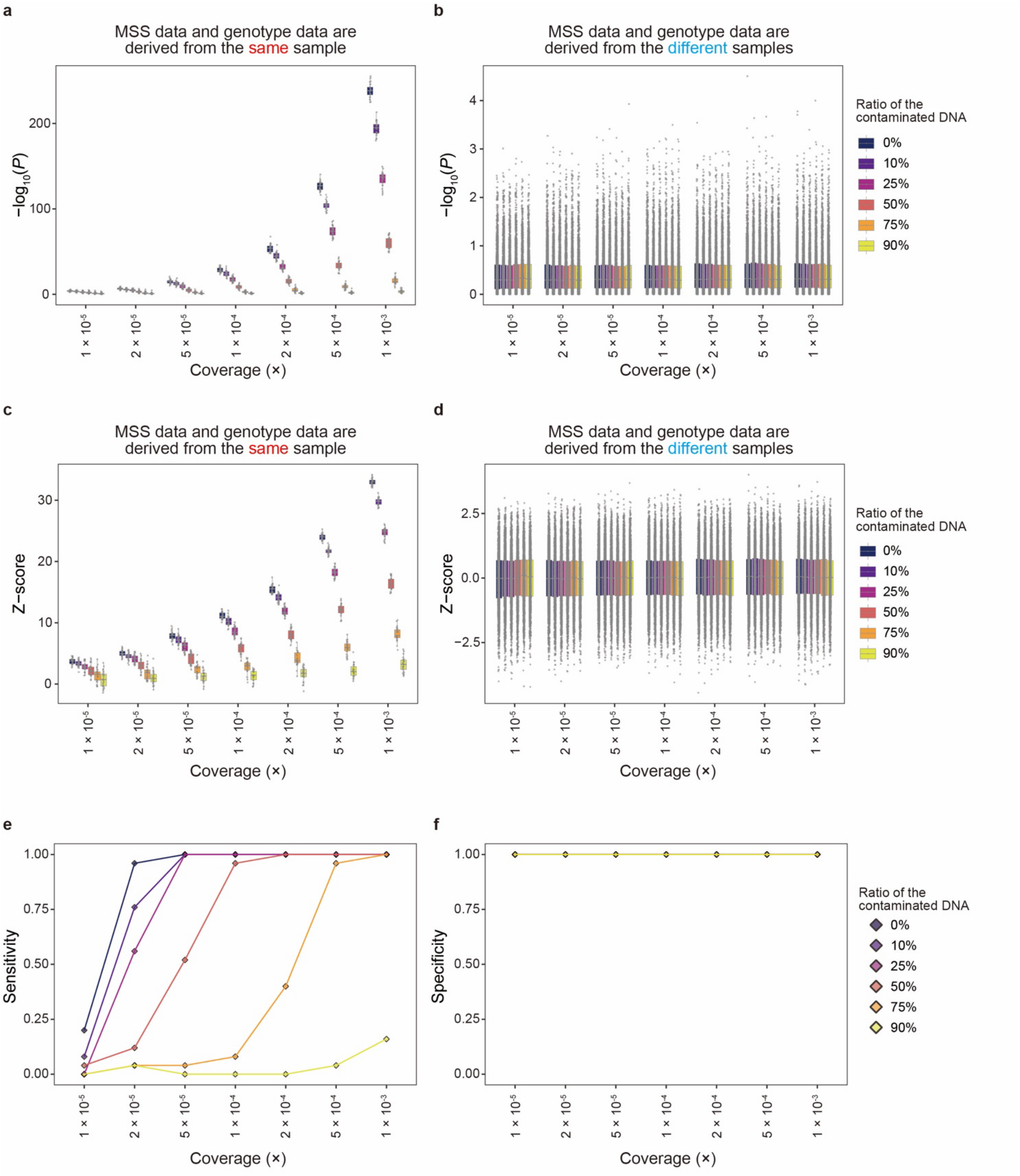

Supplementary Figure 5. Effects of the contamination of non-host human read on the efficiency of the re-identification

a-d, Box plots indicate the P-values (a,b) and Z-scores (c,d) in the re-identification analysis with simulated contaminated read data (0 ~ 90% of the reads are contaminated from the non-

host human). Cases where the metagenome data and genotype data are derived from the same sample (a,c; 5 sample × 5 seeds × 1 genotype data) and different samples (b,d; 5 sample × 5 seeds × 99 genotype data) are indicated. Boxplots indicate the median values (center lines) and IQRs (box edges), with the whiskers extending to the most extreme points within the range between (lower quantile − [1.5 × IQR]) and (upper quantile + [1.5 × IQR]). **e,f** Plots represent the sensitivity (e) and specificity (f) of the re-identification with the simulated contaminated read data. The colors of the plots indicate the ratio of the contaminated reads. IQR, interquartile ranges.

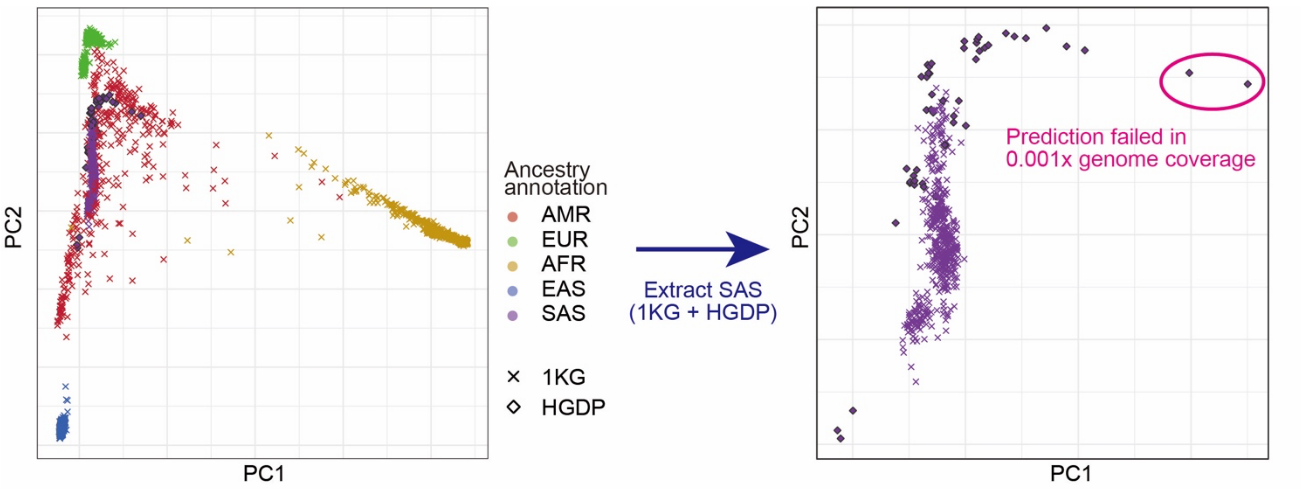

Supplementary Figure 6. Principal component analysis of the 1KG and HGDP datasets

The result of the principal component analysis of the 1KG genotype data (N = 2,504) and the SAS HGDP data used for the simulation analysis (N = 50). Only SAS populations are indicated in the right panel. 1KG, 1000 Genome Project; AFR, African; AMR, American; EAS, East Asian; EUR, European; HGDP, Human Genome Diversity Project; PC, principal components; SAS, South Asian.

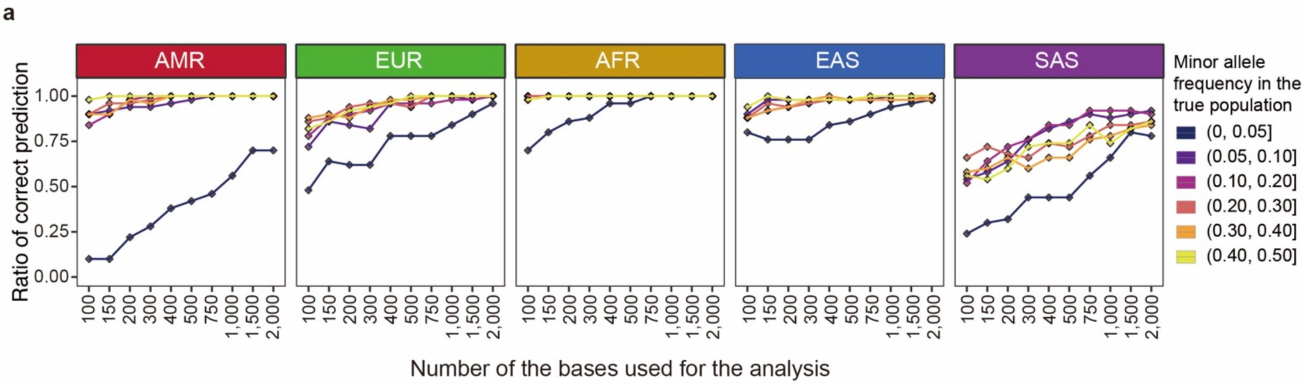

Supplementary Figure 7. Effects of the allele frequency on the performance of the ancestry prediction

Plots represent the correct prediction ratio for the ancestry prediction based on different numbers and minor allele frequencies of the SNPs. The y-axis of the plots indicates the correct prediction ratio. The x-axis of the plots indicates the number of bases used for the calculation of the likelihood scores. The colors of the plots indicate the minor allele frequency bin of the SNPs. AFR, African; AMR, American; EAS, East Asian; EUR, European; SAS, South Asian.

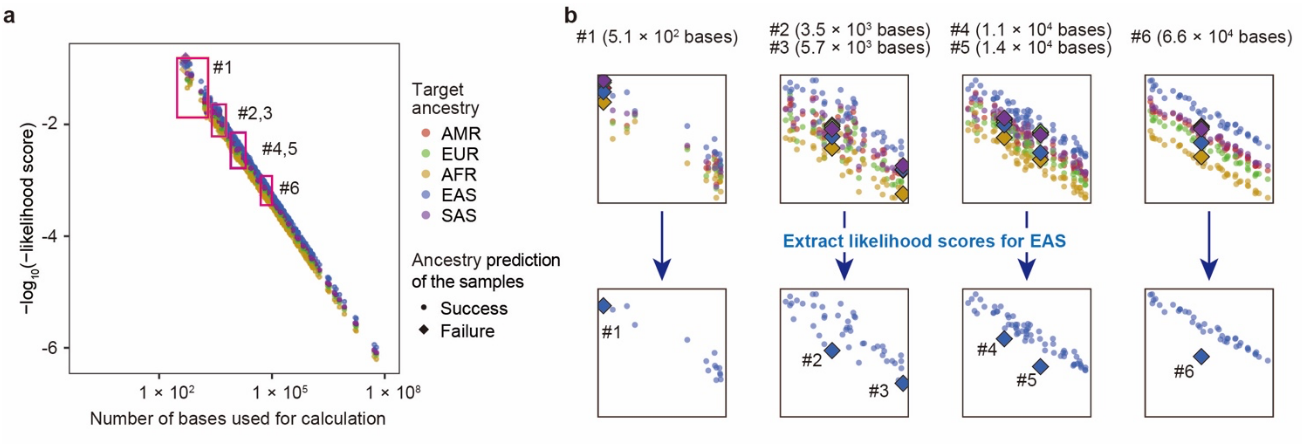

Supplementary Figure 8. Misclassified cases in the likelihood score-based ancestry prediction

The six misclassified cases are picked up from **Fig. 3b**. Four areas indicated in panel **a**, which contain the misclassified cases, are presented as larger images in panel **b**. The x-axis of the plots indicates the number of bases used for the calculation of the likelihood scores. The y-axis of the plot indicates the likelihood score. The colors of the points indicate the target ancestries and the shapes of the points indicate whether the ancestry prediction is correct or not. AFR, African; AMR, American; EAS, East Asian; EUR, European; SAS, South Asian.

When the number of bases was small, the differences in the likelihood scores between populations were not so clear. Under these circumstances, misclassification could occur as a probabilistic event (Sample #1~#3). When the number of bases was relatively large, the differences in the likelihood scores between populations were clear. However, the misclassified samples (Sample #5 and #6) had outlier scores compared to other samples, which suggested that other factors, such as contamination, could contribute to the misclassifications.

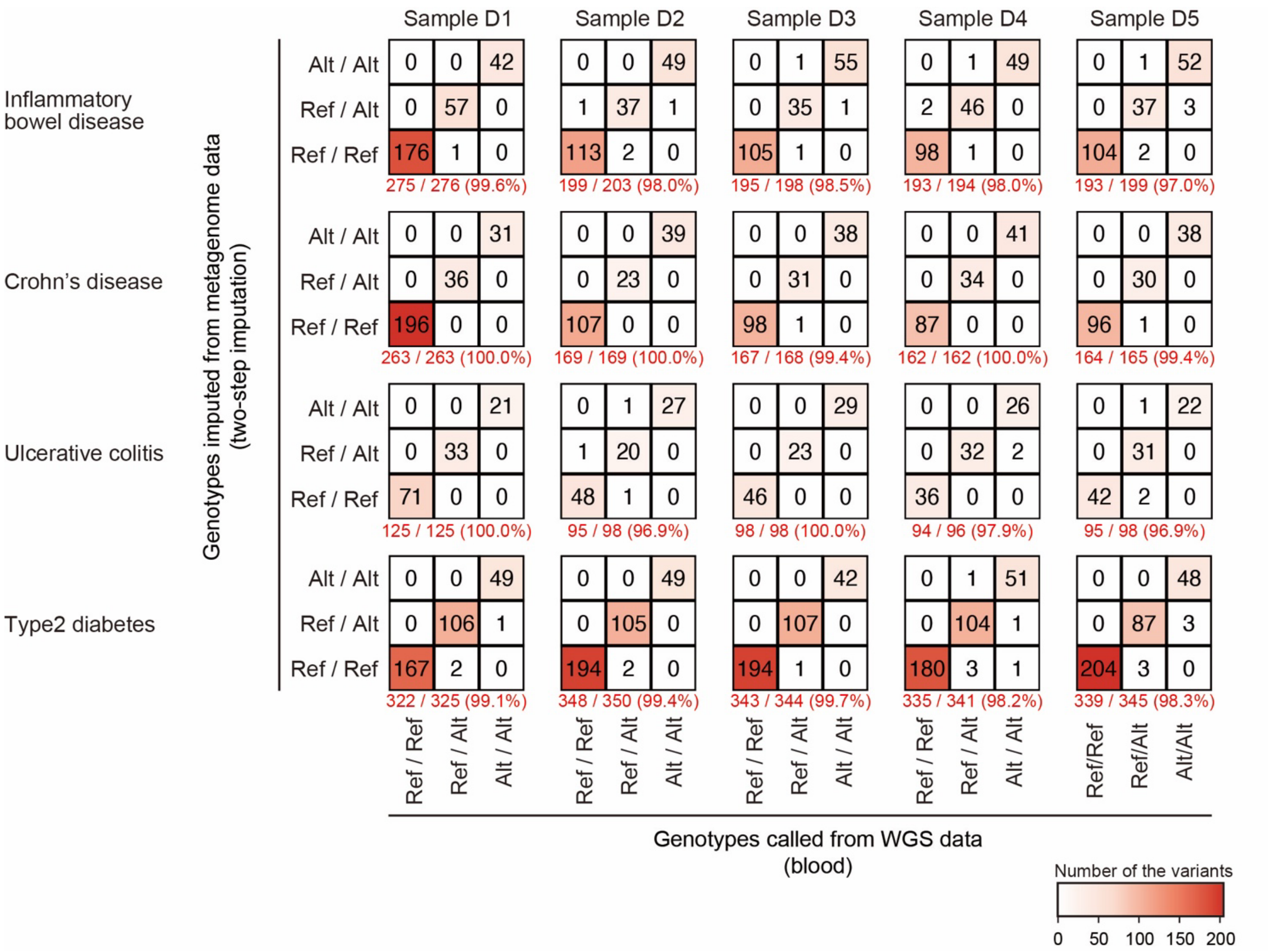

Supplementary Figure 9. Two-step genotype calling for the disease-associated SNPs from ultra-deep gut metagenome shotgun sequencing data

Tile plots indicate the concordance between the genotypes called from ultra-deep metagenome data (x-axis) and WGS data (y-axis) for the disease-associated SNPs (**Methods**). The color of the tiles indicates the number of the SNPs. Results for the 5 samples × 4 diseases are indicated. Alt, alternative allele; pLoF, putative loss of function; Ref, reference allele; SNP, single nucleotide polymorphism; WGS, whole genome sequencing.

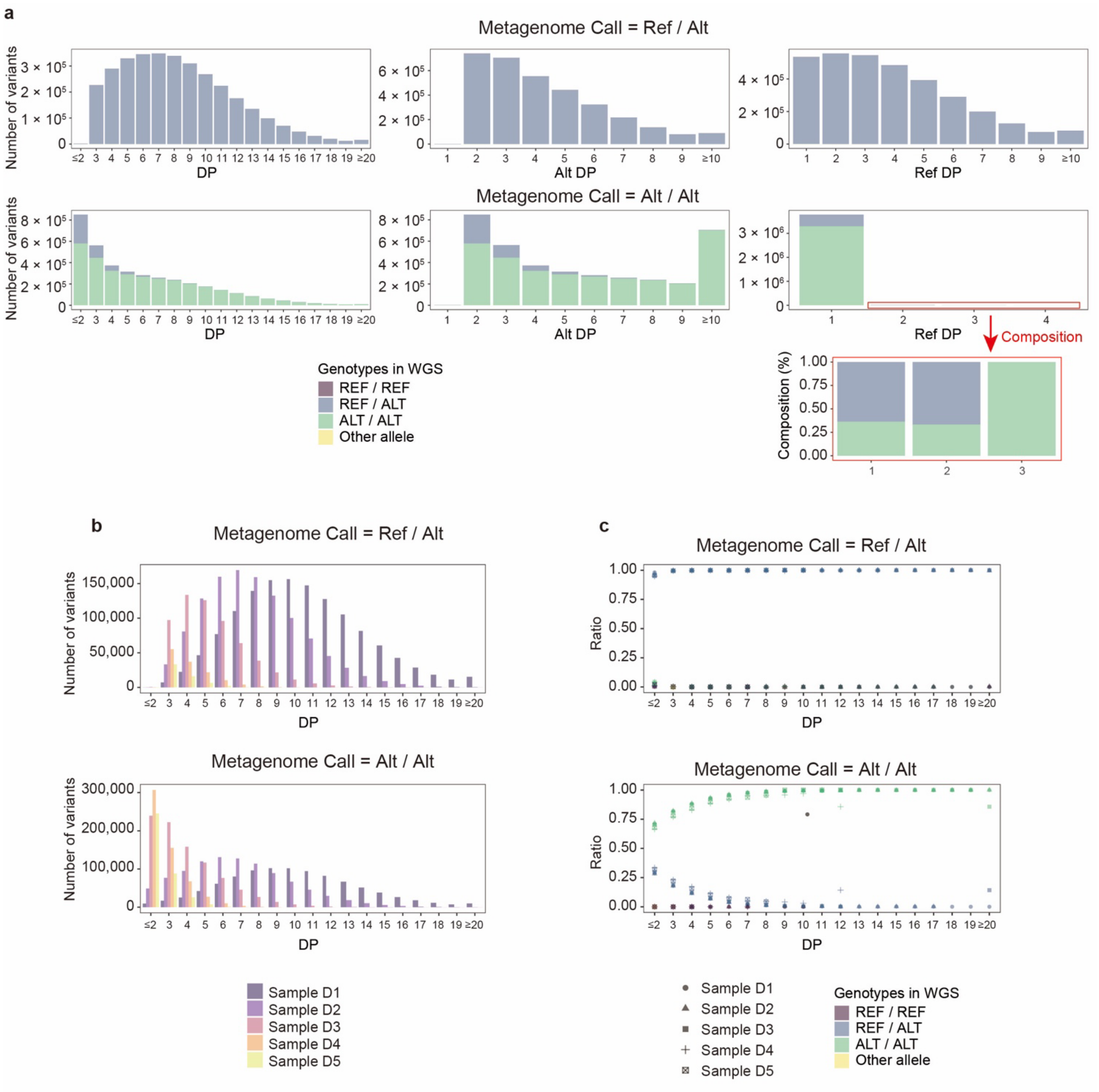

Supplementary Figure 10. Quality control of the variants called from the ultra-deep gut metagenome shotgun sequencing data without imputation

a, WGS genotypes of the variants called as heterozygous (top) and homozygous for alternative allele (bottom) in the ultra-deep gut metagenome data. The x-axis indicates the depth of all alleles (left), alternative alleles (middle), and reference alleles (right). The y-axis indicates the number of variants. The colors of the bar indicate the genotypes in the WGS. **b,** The number of variants called as heterozygous (top) and homozygous for alternative allele (bottom) in the ultra-deep gut metagenome data for each sample. The x-axis indicates the

depth. The y-axis indicates the number of variants. The colors of the bars indicate the samples. **c,** The ratio of the WGS genotypes of the variants called as heterozygous (top) and homozygous for alternative allele (bottom) in the ultra-deep gut metagenome data for each sample. The x-axis indicates the depth. The y-axis indicates the ratio of the variants. The colors of the points indicate the genotypes in the WGS. The shapes of the points indicate the samples. Alt, alternative allele; DP, depth; QC, quality control; Ref, reference allele; WGS, whole genome sequencing.

Supplementary Notes

Supplementary Note 1. Quality of human reads in the metagenome data

The human-derived reads' GC ratio and Ti / Tv ratio did not much differ from the values predicted to be acquired from the whole human genome (**Supplementary Fig. 1a,b**)[1]. In the evaluation of the read's quality, human-derived reads tended to have a greater read-through ratio and shorter length after QC than other reads (**Supplementary Fig. 1c,d**). Motivated by previous works on ancient genomes[2,3], we evaluated the non-host human-derived contamination based on the human mitochondrial DNA (mtDNA) in the gut metagenome data (**Methods**). In the simulation analysis, both of the metrics, namely the ratio of the non-major bases at polymorphic sites and major haplogroups of mtDNA estimated by mixemt[4], showed a correlation to the contamination ratio especially when there was >10× mtDNA coverage (**Supplementary Fig. 2a,b**). The ratio of the non-major bases at polymorphic sites was also affected by the mtDNA coverage, possibly due to the differences in sensitivity for detecting polymorphic sites. In the analysis with the 343 gut metagenome data, 26.8% (92 / 343) of the gut metagenome data had >10× mtDNA coverage, and 10 of these samples had a lower major haplogroup ratio than the minimum value for the simulated data with no contamination, suggesting the possible contamination of non-host human reads (**Supplementary Fig. 2c-e**).

Supplementary Note 2. Analysis with difference human reference genomes

We evaluated whether the choices of the human reference genome (GRCh38 and telomere-to-telomere reference genome chm13v2.0 [T2T-CHM13v2.0][5]) affected the prediction performance. The slight increase of the human read numbers from GRCh37 and differences in the sex-chromosomal reads between sexes were consistently observed in the GRCh38

and T2T-CHM13v2.0 (**Extended Data Fig. 2a**,**b**). The number of Y chromosomal reads in females, which should be an incorrect mapping, was decreased in the T2T-CHM13v2.0. In a logistic regression analysis, the genetic sex of 98.2% and 99.1% of the samples in the validation dataset were correctly predicted, respectively for GRCh38 and T2T-CHM13v2.0 (AUC-ROC = 0.999 and 1.000; **Extended Data Fig. 2c,d, Supplementary Table 3**). Although the difference between the reference genomes was too small to draw a definitive conclusion, the high completeness of the T2T-CHM13v2.0 might improve the prediction performance. Although the utilization of the T2T-CHM13v2.0 reference genome for the generation of the population reference panel, such as the 1KG dataset, is still underway, a future release of the T2T-CHM13v2.0 version of those data may also improve the performance of our re-identification and ancestry prediction methods.

Supplementary Note 3. Effect of allele frequency, sample size, and contamination on the performance of re-identification

We performed simulation experiments utilizing only SNP sites whose minor allele frequency (MAF) fell into specific ranges. When we used only the relatively rare SNP sites (MAF < 5%), the power of the analysis was slightly lower than the analyses with more common SNP sites (**Extended Data Fig. 4a-d**). We performed simulation experiments with a wide range of genotype data sizes (10 ~ 5,000 unrelated individuals) or significance thresholds ($P = 2.0 \times 10^{-4} \sim 2.0 \times 10^{-11}$, corresponding to the 25 metagenome data × 10 ~ 100,000,000 genotype data). The sensitivity decayed with the increase in the number of the tests due to the multiple-test burden, especially when the human read coverage was ≤0.00002×, while the type 1 error was controlled (**Extended Data Fig. 4e,f**). In the simulation analysis, 1.0×10^{-4}× human reads were sufficient to predict the matched genotype data of 25 samples from 100,000,000 individuals. In the re-identification analysis with the simulated contaminated data (**Methods**),

the contamination of the non-host human reads decreased the power of the analyses, while the null distribution of the P-values was still calibrated (**Supplementary Fig. 5a-d**). When the human reads coverage was ≥$2.0 \times 10^{-4}\times$, the sensitivity of re-identification was 100% at the threshold of $P < 2.0 \times 10^{-5}$ when 50% of the reads were derived from non-host humans (**Supplementary Fig. 5e,f**).

Supplementary Note 4. Simulation analysis for the ancestry prediction

We simulated the human reads in the gut metagenome data of the five ancestries (AMR, EUR, AFR, EAS, and SAS) by down-sampling the WGS data from the Human Genome Diversity Project (HGDP)[6] and evaluated the performance of the likelihood score-based predictions (**Supplementary Table 6, Methods**). When the number of bases used for the calculation of the likelihood scores were large, the likelihood scores for the true ancestries were higher than for the other ancestries, resulting in high true prediction ratios (true prediction ratio was 0.99 with 0.001× human genome coverage; mean number of the bases and reads were 7.7×10^4 and 1.1×10^4, respectively; **Extended Data Fig. 7a**,**b, Supplementary Table 7**). In contrast, the differences in the likelihood scores between true ancestries and other ancestries were not clear when the numbers of the bases were small, resulting in a poor true prediction ratio (true prediction ratio was 0.73 with 0.00001× human genome coverage; mean number of the bases and reads was 7.7×10^2 and 1.1×10^2, respectively). Ancestry prediction of the SAS subjects was relatively difficult and they tended to be misclassified to AMR and EUR, especially when the number of the bases was small (**Extended Data Fig. 7c**). This could be because the genetic diversity of the SAS population was not fully reflected in the 1KG data[7], which were also seen as a difference between the SAS subjects in the 1KG and HGDP in a PCA space (**Supplementary Fig. 6**). We performed simulation experiments by using only SNP sites whose minor allele frequency (MAF) in the

targeted population was within specific ranges. As in the case of the re-identification analysis, the prediction accuracy was relatively low when we used only the relatively rare SNP sites (MAF < 5%; **Supplementary Fig. 7**).

Supplementary Note 5. Utilization of the animal-derived reads in the gut metagenome

Stool samples have been rarely recognized as a useful source of germ line genomes in humans, but are often seen in wild and domestic animals. For example, the small amount of host reads in the stool metagenome data (e.g. 0.09 ~ 3.13% in Ang et al[8]) were utilized to recover mitochondrial genome sequences of wild primates, for which invasive sample collection is difficult. It was also reported that the ratio of host reads could be larger and recovery of host genome information might be more efficient depending on the target animals and sample collection methods[9,10]. Recently, the utility of two-step genotype imputation from the host reads in gut metagenome data was demonstrated in chicken and it might contribute to the cost-efficient analysis of the host–microbiome interaction[11].

References for Supplementary Notes

1. Romiguier, J., Ranwez, V., Douzery, E. J. P. & Galtier, N. Contrasting GC-content dynamics across 33 mammalian genomes: Relationship with life-history traits and chromosome sizes. *Genome Research* (2010) doi:10.1101/gr.104372.109.
2. Cooke, N. P. *et al.* Ancient genomics reveals tripartite origins of Japanese populations. *Science Advances* 7, eabh2419.
3. Renaud, G., Slon, V., Duggan, A. T. & Kelso, J. Schmutzi: estimation of contamination and endogenous mitochondrial consensus calling for ancient DNA. *Genome Biology* 16, 224 (2015).
4. Vohr, S. H. *et al.* A phylogenetic approach for haplotype analysis of sequence data from complex mitochondrial mixtures. *Forensic Science International: Genetics* 30, 93–105 (2017).

5. Nurk, S. *et al.* The complete sequence of a human genome. *Science* 376, 44–53 (2022).
6. Bergström Anders *et al.* Insights into human genetic variation and population history from 929 diverse genomes. *Science* 367, eaay5012 (2020).
7. Auton, A. *et al.* A global reference for human genetic variation. *Nature* 526, 68–74 (2015).
8. Ang, A. *et al.* Faecal DNA to the rescue: Shotgun sequencing of non-invasive samples reveals two subspecies of Southeast Asian primates to be Critically Endangered species. *Scientific Reports* 10, 9396 (2020).
9. de Flamingh, A. *et al.* Non-invasive fecal DNA yields whole genome and metagenomic data for species conservation. *bioRxiv* 2022.08.16.504190 (2022) doi:10.1101/2022.08.16.504190.
10. Taylor, R. S. *et al.* Whole genome sequences from non-invasively collected caribou faecal samples. *Conservation Genetics Resources* 14, 53–68 (2022).
11. Marcos, S., Parejo, M., Estonba, A. & Alberdi, A. Recovering High-Quality Host Genomes from Gut Metagenomic Data through Genotype Imputation. *Advanced Genetics* n/a, 2100065 (2022).
