# Supplementary material for: Reconstruction of the personal information from human genome reads in gut metagenome sequencing data
Source: Nat Microbiol. 2023 May 15;8(6):1079–94. doi: 10.1038/s41564-023-01381-3 (PMC10234815; doi:10.1038/s41564-023-01381-3)
Supplement: Supplementary file 2 — Reporting Summary [file 41564_2023_1381_MOESM2_ESM.pdf]

nature portfolio

Corresponding author(s): Yoshihiko Tomofuji, Yukinori Okada

Last updated by author(s): Mar 2, 2023

# Reporting Summary

Nature Portfolio wishes to improve the reproducibility of the work that we publish. This form provides structure for consistency and transparency in reporting. For further information on Nature Portfolio policies, see our Editorial Policies and the Editorial Policy Checklist.

## Statistics

For all statistical analyses, confirm that the following items are present in the figure legend, table legend, main text, or Methods section.

| n/a | Confirmed | |
|---|---|---|
| ☐ | ☒ | The exact sample size (*n*) for each experimental group/condition, given as a discrete number and unit of measurement |
| ☐ | ☒ | A statement on whether measurements were taken from distinct samples or whether the same sample was measured repeatedly |
| ☐ | ☒ | The statistical test(s) used AND whether they are one- or two-sided<br>*Only common tests should be described solely by name; describe more complex techniques in the Methods section.* |
| ☒ | ☐ | A description of all covariates tested |
| ☐ | ☒ | A description of any assumptions or corrections, such as tests of normality and adjustment for multiple comparisons |
| ☐ | ☒ | A full description of the statistical parameters including central tendency (e.g. means) or other basic estimates (e.g. regression coefficient) AND variation (e.g. standard deviation) or associated estimates of uncertainty (e.g. confidence intervals) |
| ☐ | ☒ | For null hypothesis testing, the test statistic (e.g. *F*, *t*, *r*) with confidence intervals, effect sizes, degrees of freedom and *P* value noted<br>*Give P values as exact values whenever suitable.* |
| ☒ | ☐ | For Bayesian analysis, information on the choice of priors and Markov chain Monte Carlo settings |
| ☒ | ☐ | For hierarchical and complex designs, identification of the appropriate level for tests and full reporting of outcomes |
| ☒ | ☐ | Estimates of effect sizes (e.g. Cohen's *d*, Pearson's *r*), indicating how they were calculated |

*Our web collection on statistics for biologists contains articles on many of the points above.*

## Software and code

Policy information about availability of computer code

| | |
|---|---|
| Data collection | The sequence reads were converted to the FASTQ format using bcl2fastq (version 2.19) |
| Data analysis | Annovar (Mon, 8 Jun 2020), bcftools (version 1.15.1), beagle4.1 (27Jan18), beagle5.1 (18May20), bedtools (version 2.29.2), bowtie2 (version 2.3.5.1), eigenstrat (version 6.1.4), fastqc (version 0.11.9), GATK (v3.8 and v4.1.7), IGV (version 2.12.2), minimac4 (version 1.0.1), mixemt (v0.2), Picard (version 2.22.8), plink (version 1.90b4.4), python3 (version 3.7.6), R (version 4.0.1), samtools (version 2.3.5.1), scikit-learn (version0.22.1), shapeit (version 4.2.1), tidyverse (version 1.3.0), Trimmomatic (version 0.39), VEP (version 102) were used for analyses. Scripts used for analyses will be available on github (https://github.com/ytomofuji/Human_reads_in_metagenome). |

For manuscripts utilizing custom algorithms or software that are central to the research but not yet described in published literature, software must be made available to editors and reviewers. We strongly encourage code deposition in a community repository (e.g. GitHub). See the Nature Portfolio guidelines for submitting code & software for further information.

## Data

Policy information about availability of data

All manuscripts must include a data availability statement. This statement should provide the following information, where applicable:

- Accession codes, unique identifiers, or web links for publicly available datasets
- A description of any restrictions on data availability
- For clinical datasets or third party data, please ensure that the statement adheres to our policy

The metagenome shotgun sequencing data generated in this study (multi-ancestry dataset, ultra-deep sequencing dataset, and dataset generated with DNeasy PowerSoil kit) are available at the Japanese Genotype-Phenotype Archive (JGA) under accession number JGAS000600. The SNP array and WGS data for the human blood used in this study are available at the European Genome-Phenome Archive (EGA) under accession number EGAS00001007027. The Japanese metagenome

shotgun sequencing data derived from the previous studies (dataset 1-3 and validation dataset) are available at the JGA under accession numbers JGAS000260, JGAS000316, JGAS000531, and JGAS000415, respectively. The metagenome shotgun sequencing data derived from Zhu et al. are available at the European Nucleotide Archive (ENA) database under accession number PRJEB29127. The metagenome shotgun sequencing data derived from Dhakan et al. are available at the Sequence Read Archive (SRA) database under accession number PRJNA397112. The metagenome shotgun sequencing data derived from Karlsson et al. are available at the SRA database under accession number PRJEB1786. The genotype data of the BBJ 2nd cohort are available at JGA under accession number JGAS000412.

# Field-specific reporting

Please select the one below that is the best fit for your research. If you are not sure, read the appropriate sections before making your selection.

☒ Life sciences ☐ Behavioural & social sciences ☐ Ecological, evolutionary & environmental sciences

For a reference copy of the document with all sections, see nature.com/documents/nr-reporting-summary-flat.pdf

# Life sciences study design

All studies must disclose on these points even when the disclosure is negative.

**Sample size**

We examined 343 individuals with available metagenome shotgun sequencing data and genotype data.
We also examined 113 and 73 individuals for the validation of the sex-prediction analysis and ancestry-prediction analysis, respectively.
No statistical methods were used to pre-determine sample sizes but our sample sizes are similar to those reported in previous publications.

**Data exclusions**

No samples were excluded from analyses.

**Replication**

In this paper, we used three different datasets to confirm the reproducibility of the results for key findings. In addition, analysis with other datasets was also performed for sex prediction and ancestry prediction. Evaluation of the technical effects (DNA extraction methods) was also performed throughout the paper.

**Randomization**

Not applicable. This study is an observational study and does not involve intervention.

**Blinding**

We did not apply binding of the samples because no intervention was conducted in our study.

# Reporting for specific materials, systems and methods

We require information from authors about some types of materials, experimental systems and methods used in many studies. Here, indicate whether each material, system or method listed is relevant to your study. If you are not sure if a list item applies to your research, read the appropriate section before selecting a response.

## Materials & experimental systems

| n/a | Involved in the study | |
|---|---|---|
| ☒ | ☐ | Antibodies |
| ☒ | ☐ | Eukaryotic cell lines |
| ☒ | ☐ | Palaeontology and archaeology |
| ☒ | ☐ | Animals and other organisms |
| ☐ | ☒ | Human research participants |
| ☒ | ☐ | Clinical data |
| ☒ | ☐ | Dual use research of concern |

## Methods

| n/a | Involved in the study | |
|---|---|---|
| ☒ | ☐ | ChIP-seq |
| ☒ | ☐ | Flow cytometry |
| ☒ | ☐ | MRI-based neuroimaging |

## Human research participants

Policy information about studies involving human research participants

**Population characteristics**

343 + 113 Japanese subjects for whom gut metagenome shotgun sequencing data were performed in previous studies were included in this study. We also included 73 individuals living in Japan or India. The main, validation, and multi-ancestry dataset included 343, 113, and 73 subjects; 196, 65, and 25 females, and 147, 48, and 48 males. Ranges of their age were 20-88 (mean 42.2, SD 17.3), 20-81 (mean 39.1, SD 17.3), and 20-61 (mean 30.5, SD 7.9). Further information can be found in Supplementary Table 1.

**Recruitment**

The subjects were enrolled at the Osaka University and related medical institutions or recruited in Delhi area. Although all healthy participants were recruited based on voluntary participation in this study, the effect of the self-selection bias would be minimal because we did not perform any case–control comparison.

**Ethics oversight**

The study protocol was approved by the ethics committees of Osaka University and related medical institutions and the Translational Health Science and Technology Institute (Faridabad)

Note that full information on the approval of the study protocol must also be provided in the manuscript.
